# Determination of Toxic Pyrrolizidine Alkaloids in Traditional Chinese Herbal Medicines by UPLC-MS/MS and Accompanying Risk Assessment for Human Health

**DOI:** 10.3390/molecules26061648

**Published:** 2021-03-16

**Authors:** Junchi Wang, Meng Zhang, Lihua Chen, Yue Qiao, Siqi Ma, Dian Sun, Jianyong Si, Yonghong Liao

**Affiliations:** 1 The Key Laboratory of Bioactive Substances and Resources Utilization of Chinese Herbal Medicine, Ministry of Education, Institute of Medicinal Plant Development, Chinese Academy of Medical Sciences & Peking Union Medical College, Beijing 100193, China; jcwang@implad.ac.cn (J.W.); mandy6378@126.com (M.Z.); lihuachen0706@163.com (L.C.); MOON100107qy@163.com (Y.Q.); siqimazry@163.com (S.M.); dasun@implad.ac.cn (D.S.); 2 State Key Laboratory of Natural and Biomimetic Drugs, School of Pharmaceutical Sciences, Peking University, Beijing 100191, China

**Keywords:** pyrrolizidine alkaloids, Chinese herbal medicines, estimated daily intake, Margin of exposure, risk assessment

## Abstract

Pyrrolizidine alkaloids (PAs) are a class of natural toxins with hepatotoxicity, genotoxicity and carcinogenicity. They are endogenous and adulterated toxic components widely found in food and herbal products. In this study, a sensitive and efficient ultra-high performance liquid chromatography-tandem mass spectrometry (UPLC-MS/MS) method was used to detect the PAs in 386 kinds of Chinese herbal medicines recorded in the Chinese Pharmacopoeia (2020). The estimated daily intake (EDI) of 0.007 μg/kg body weight (bw)/day was adopted as the safety baseline. The margin of exposure (MOE) approach was applied to evaluate the chronic exposure risk for the genotoxic and carcinogenic potential of PAs. Results showed that PAs was detected in 271 out of 386 samples with a content of 0.1–25,567.4 μg/kg, and there were 20 samples with EDI values above the baseline, 0.007 μg/kg bw/day. Beyond that, the MOE values for 10 out of 271 positive samples were below 10,000. Considering the actual situation, Haber’s rule was used to assume two weeks exposure every year during lifetime, and still the MOE values for four out of 271 positive samples were under 10,000, indicating these products may have potential health risk. The developed method was successfully applied to detect the PAs-containing Chinese herbal medicines. This study provides convincing data that can support risk management actions in China and a meaningful reference for the rational and safe use of Chinese herbal medicines.

## 1. Introduction

Pyrrolizidine alkaloids (PAs) are widespread secondary plant metabolites produced as defense against herbivores [1]. It is reported that more than 660 PAs and their *N*-oxides have been identified in over 6000 plants distributed in many geographical regions worldwide, accounting for 3% of all flowering plants [2,3], and about half of these phytochemicals have been reported to be hepatotoxic in human and livestock [4]. The ability to produce PAs has been found in representatives of Asteraceae, Boraginaceae, and Fabaceae families [5,6].

PAs are composed of a 1-hydroxymethyl pyrrolizidine (necine base) and aliphatic monocarboxylic or dicarboxylic acids (necine acids), and can be classified into monoesters or diesters according to the esterification with one or both hydroxyl groups. Esterification with two carboxyl groups of a dicarboxylic acid can result in a cyclical diester. A distinction can be made among 1,2-unsaturated PAs of the retronecine-, heliotridine- or otonecine-type, and the necine bases of retronecine and heliotridine are diastereomers due to the different configurations at C-7 [7]. Hepatotoxicity is considered to be enhanced by esterification of the hydroxyl groups at C-7 [8,9]. Therefore, cyclic diesters have the strongest toxic effects, followed by monoesters and open-chain diesters of 1,2-unsaturated PAs [1,10].

**Figure 1:**
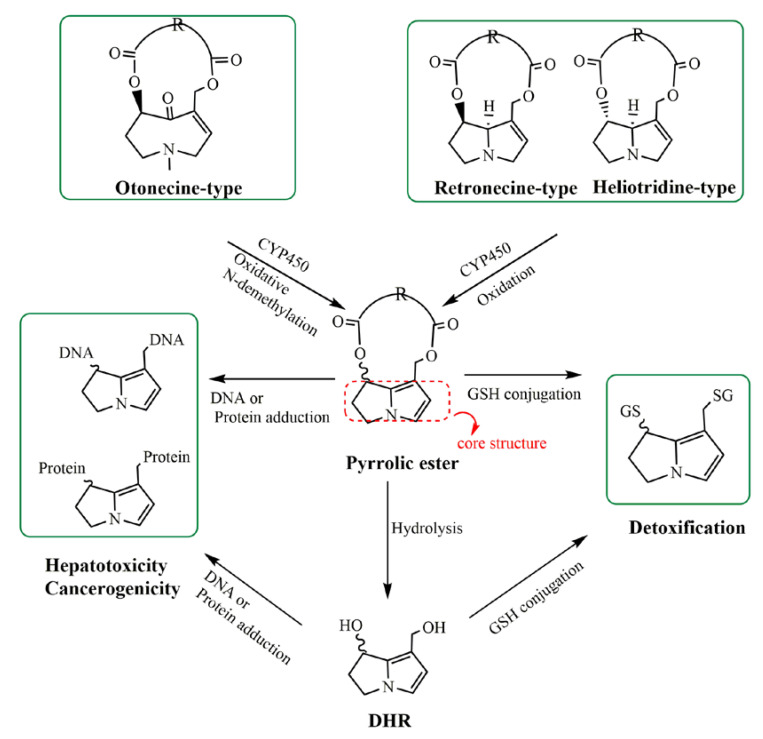
Metabolic activation of retronecine-, heliotridine-, and otonecine-type pyrrolizidine alkaloids (PAs) leading to hepatotoxicity and tumorigenicity, detoxification.

PA poisoning cases have been extensively reported [11,12,13] and long-term exposure to PAs can cause hepatic veno-occlusive disease (HVOD) and human hepatic sinusoid occlusion syndrome (HSOS) [14]. It is generally accepted that PAs themselves are not toxic and need hepatic cytochrome P450 monooxygenases (CYP450, mainly CYP3A4) to mediate the metabolic activation. As illustrated in Figure 1, three types PAs can be converted into 6,7-dihydro-7-hydroxy-1-hydroxymethyl-5*H*-pyrrolizidine (DHP) esters, which can be further converted into DHP by hydrolysis [1,15]. The pyrrolic metabolites, DHP esters and DHP, are highly reactive bifunctional alkylating agents, once formed can rapidly bind to nucleophilic groups in cellular proteins and DNA, including DNA binding, DNA cross-linking, DNA-protein cross-linking [7,15], and then may result in the acute and chronic hepatotoxicity, genotoxicity and carcinogenicity. Similar to DHP esters, DHP can also bind with GSH, DNA or proteins to generate pyrrole-GSH conjugates, pyrrole-protein adducts or pyrrole-DNA adducts, respectively, leading to detoxification, hepatotoxicity or carcinogenicity [7,16].

Herbal medicines were widely used to treat disease and maintain human health for thousands of years in many countries and have become more and more popular [17]. In recent years, studies have shown that PAs components have been detected in some common Chinese herbal medicines [18,19,20]. Although some PAs have been analyzed in the absence of relevant reference materials, their exact content remains to be investigated. In addition, the Chinese Pharmacopoeia (2020) clarifies that some Chinese herbal medicines do contain PAs, but only limited PAs have been strengthened the toxicity control, and the vast majority of Chinese herbal medicines (including decoction pieces and preparations) have not established the standard of such ingredients, leaving certain potential threats in clinical use and daily application. However, to our best knowledge, the recent safety control study for PAs just focus on limited variety of Chinese medicines [21,22] and there is no official statement on PAs intake in China as yet. Accordingly, in order to verify the presence and content of natural toxic PAs in herbal medicines of Chinese Pharmacopoeia (2020), it is urgent to establish a feasible quantitative analysis method and assess the risk based on the PAs content.

Given that 1,2-unsaturated PAs are reported to be genotoxic and carcinogenic, health risks were assessed by using the margin of exposure (MOE) approach, which is regarded as the most suited procedure [23]. MOE values below 10,000 indicate that there might be a potential concern for human health. To date, the MOE is calculated on the basis of a BMDL_10_ (benchmark dose lower confidence limit for 10% extra risk on tumor formation above background levels) of 237 µg/kg bw/day originating from a chronic exposure experiment with riddelliine in rats [24]. The CONTAM Panel concluded that the data from experimental animals are relevant to humans and the carcinogenicity data provided the most suitable basis for the risk characterization.

The aim of this study is to quantify the PAs total content and conduct the risk assessment in 386 commonly used Chinese herbal medicines recorded in Chinese Pharmacopoeia (2020). We aim to provide an effective tool for the qualification control and quantitative analysis of PAs in Chinese herbal medicines and scientific reference to guide the rational and safe utilization of these herbs for human health.

## 2. Results

### 2.1. Pretreatment Method Development

#### 2.1.1. Extraction Optimization

**Figure 2:**
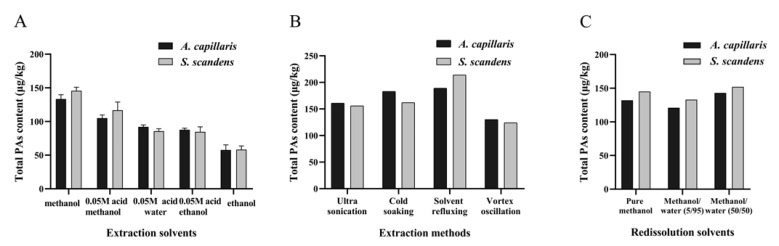
Effect of five different extraction solvents (*n* = 3) (**A**), four different extraction methods (**B**), and three different redissolution solvents (**C**) on the total PAs content of herbal medicines in *A. capillaris* (dark gray bars) and *S. scandens* (light gray bars).

To compare extraction conditions, raw extracts of *Artemisia capillaris* (*A. capillaris*) and *Senecio scandens* (*S. scandens*) samples were prepared. Each test with five different extraction solvents, MeOH, 0.05 M sulfuric acid in MeOH, 0.05 M sulfuric acid water, ethanol and 0.05 M sulfuric acid in ethanol, was repeated three times. As shown in Figure 2A (more details see Tables S4 and S5), MeOH had the highest PAs extraction efficiency at 133.4 µg/kg and 145.7 µg/kg respectively. Accordingly, pure MeOH was selected as extraction solvents. In the extraction aids test, it was interest to find that solvent refluxing had the highest PAs extraction efficiency, sonication and cold soaking came to the next, and vortex oscillation was the last one (Figure 2B). While considering the instability of pyrrolizidine alkaloid *N*-oxides (PANOs) in high temperature, as well as the quick and simple operation characteristics, ultrasonication (100 W, 40 KHZ, 30 min) was adopted as the final extraction mode.

Furthermore, different ratios of the elution solvent NH_4_OH/MeOH (3:17→1:3, *v*/*v*) were tested, and the best ratio was 1:3 (*v*/*v*). Besides, three different ratios of redissolution solvents were tested: pure MeOH, MeOH/water (50/50, *v*/*v*) and MeOH/water (5/95, *v*/*v*), results were shown in Figure 2C, the MeOH/water (50/50, *v*/*v*) were selected as the final redissolution solvents with the high recoveries and solubility.

#### 2.1.2. Purification Optimization

**Figure 3:**
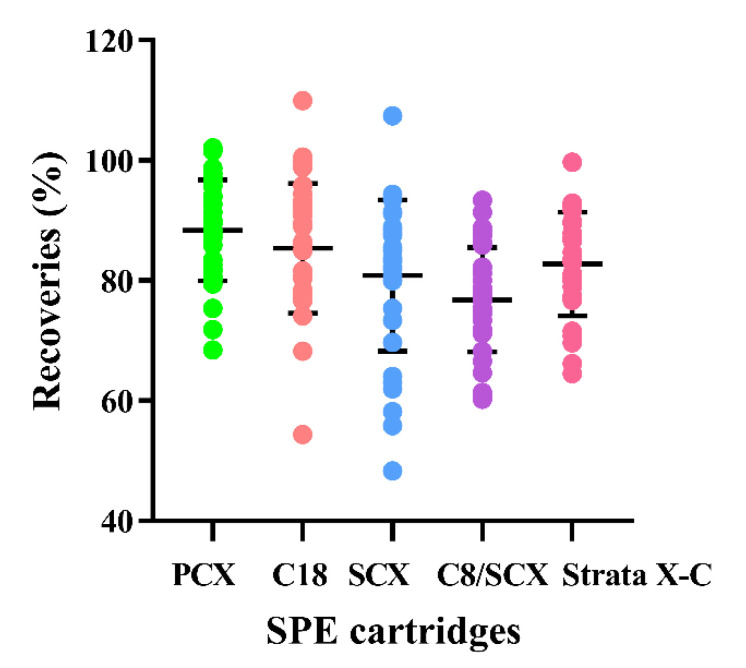
Effects of five different solid phase extration (SPE) cartridges on the recoveries of 34 PAs compounds.

Five different kinds of solid phase extration (SPE) cartridges (500 mg/6 mL) were investigated: Cleanert PCX, SCX, C18, C8/SCX (all from Agela Technologies, Tianjin, China) and Strata-X-C (Phenomenex, Guangzhou, China), and the recoveries were tested by adding a matrix free mixed acid standard solution to each of the five SPE cartridges, the results were shown in Figure 3 (more details see Table S6). 

Overall, Cleanert PCX, a mixed-mode sorbent which provided dual retention modes of reversed-phase and cation-exchange, was found to be the most appropriate clean-up cartridge. 

### 2.2. UPLC-MS/MS Method Development

**Figure 4:**
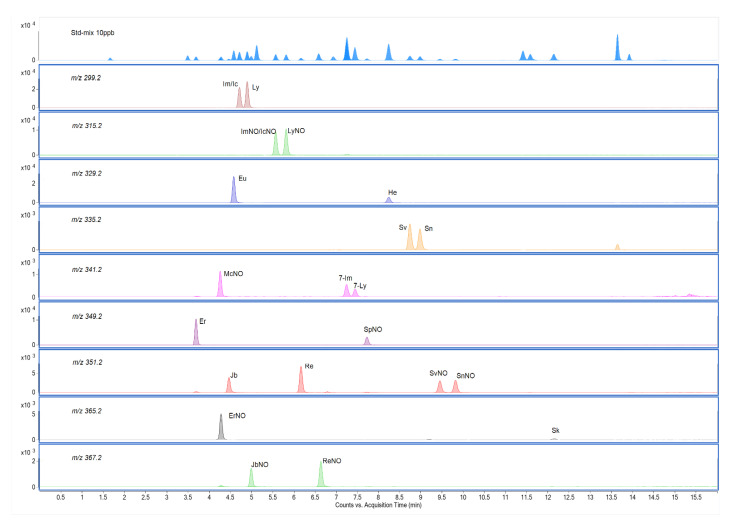
Multiple reaction monitoring (MRM) chromatograms of nine sets of isomers with a mixed standard of PAs (10 μg/kg) by liquid chromatography-tandem mass spectrometry (LC-MS/MS). For each pair the molecular mass and compound name (Im: intermedine; Ic: indicine; Ly: lycopsamine; ImNO: intermedine *N*-oxide; IcNO: indicine *N*-oxide; LyNO: lycopsamine *N*-oxide; Eu: europine; He: heliotrine; Sv: senecivernine; Sn: senecionine; McNO: monocrotaline *N*-oxide; 7-Im: 7-acetylintermedine; 7-Ly: 7-acetyllycopsamine; Er: erucifoline; SpNO: seneciphylline *N*-oxide; Jb: jacobine; Re: retrorsine; SvNO: senecivernine *N*-oxide; SnNO: senecionine *N*-oxide; ErNO: erucifoline *N*-oxide; JbNO: jacobine *N*-oxide; ReNO: retrorsine *N*-oxide) were shown.

Under optimum conditions, 18 PAs and 14 PANOs, including nine sets of isomers were separated completely among these 34 compounds. Only two pairs of stereoisomers (intermedine and indicine, intermedine *N*-oxides and indicine *N*-oxides) were not separated, but the two unseparated PAs had the same response value as their corresponding isomers. PAs were quantitatively determined in multiple reaction monitoring (MRM) acquisition mode. The MRM chromatograms of these isomers were shown in Figure 4. 

As for the MS^2^ spectra, the fragment ions of *m*/*z* 120, 118, 138, 136, 150 and 168 are characteristic for PA free bases related to necine, while product ions of *m*/*z* 120 and 138 are typical of retronecine-type and heliotridine-type PAs, and *m*/*z* 168 and 150 for otonecine otonecine-type. The most intense fragment with most intense signal of each PAs was used as quantification ion and another transition as confirmation ion. The MS conditions of 34 PAs and the corresponding *N*-oxides are listed in Table S1.

### 2.3. Method Validation

#### 2.3.1. Sensitivity, Linearity, Limits of Detection (LOD) and Limits of Quantification (LOQ)

Due to the difference in concentration between different samples, the calibration curve of a wide concentration range from 0.1 ng/mL to 500 ng/mL for the analyses of PAs in herbal plants was applied. Table S7 shows the regression equation and the linear regression coefficients for the PAs, all 32 PA standards with the correlation coefficient (R^2^) values being >0.99, indicating the excellent linearity. As shown in Table S7, The LOD of PAs with a S/N ratio of >3 ranged from 0.01~0.2 µg/kg, whereas the LOQ with a S/N ratio of >10 between 0.1 and 0.5 µg/kg herbs, indicating high sensitivity for the determination of trace PAs in these Chinese herbal medicines. 

#### 2.3.2. Precision and Recovery

The samples were analyzed with the same instrument and the same operator. As shown in Table S7, results show that the intraday and interday precision expressed by RSD (%) were less than 8% and no significant distinction was found between them, the tolerance of repeatability and stability were tested by repeated injecting 100 ng/mL standard solution (*n* = 6) and behaved well with the RSD < 5%. The results suggested that the present method has acceptable accuracy and precision.

### 2.4. PA Concentrations in Chinese Herbal Medicines

**Figure 5:**
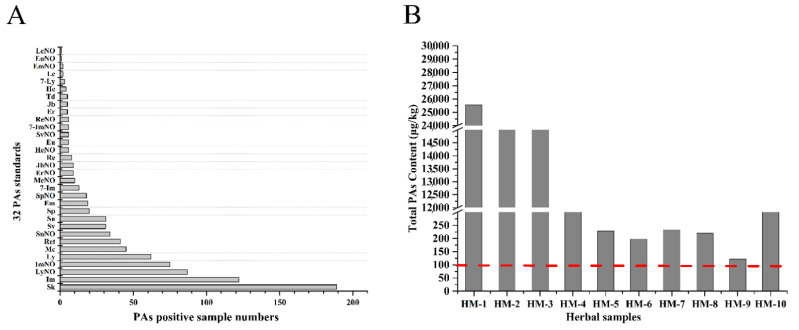
The distribution of 32 PAs standards in 271 PA-positive herbal medicinal materials (**A**), senkirkine, intermedine and lycopsamine-*N*-oxide were the top three most frequently found PAs, and (**B**) showed top ten herbal medicines with total PAs content above 100 μg/kg, namely HM-1(*Arnebia euchroma*): 25567.4 μg/kg; HM-2 (*Tussilago farfara*): 17,600.1 μg/kg; HM-3 (*Eupatorium fortune*): 16,943.6 μg/kg; HM-4 (*Eupatorium lindleyanum*): 1863.6 μg/kg; HM-5 (*Senecio scandens*): 233.6 μg/kg; HM-6 (*Laggera pterodonta*): 229.0 μg/kg; HM-7 (*Artemisia scoparia*): 220.6 μg/kg; HM-8 (*Cassia angustifolia*): 198.5 μg/kg; HM-9 (*Euphorbia hirta*): 121.9 μg/kg; HM-10 (*Gynura japonica*): 531.0 μg/kg.

Based on the above-established pretreatment method and UPLC-MS/MS analysis method, 386 kinds of Chinese herbal medicines were detected and analyzed, including 128 kinds of classical Chinese medicinal materials and 81 kinds of medicinal and food dual-purpose materials. PAs were detected in 271 out of 386 herbal medicine samples (more than 70%), which was significantly higher than a study conducted on the spices and culinary herbs from Asia (36 positive samples of 71, 50.7%) [25], but lower than another study on herbs and spices (17 positive samples of 17, 100%) [26]. The detected PAs content was not less than 0.1 μg/kg. Senkirkine, intermedine and lycopsamine-*N*-oxide were the top three most frequently found PAs (see Figure 5A). Among these, the most widely distributed PAs was senkirkine, accounting for 68% (189/271) of the positive medicinal materials, which was also one of the most frequently occurring individual PA/PANO in the spices and culinary herbs from Asia in a previous study [25]. However, different from this, europine and europine *N*-oxide were the most widely distributed PAs in Mediterranean herbs mixes [26]. Intermedine came to the next in our study, accounting for 44% (122/271). It was interest to note that most of the high-content PAs samples were distributed in Compositae (38/271), Leguminosae (33/271), Ranunculaceae (11/271) and Labiatae (9/271), and this finding was in general agreement with previous reports [5,6,25]. Among them, there were 10 samples that PAs contents are more than 100 μg/kg, ranged from 121.9 to 25567.4 μg/kg. The highest PA level was found in sample HM-1, *Arnebia euchroma* (*A. euchroma*, see Figure 5B).

### 2.5. Risk Assessment of Chinese Herbal Medicines Based on PAs Levels

#### 2.5.1. Acute Exposure Scenario

The related regulations set by the European Medicines Agency (EMA) combined with the Committee on Toxicity of Chemicals in Food, Consumer Products and Environment (COT) and German Federal Institute for Risk Assessment (BfR) show that samples with estimated daily intake (EDI) values below the 0.007 μg/kg bw/day are unlikely to raise concern [27], meanwhile, European Food Safety Authority (EFSA) has reported several acute/short-term adverse exposure cases in human at the dose range of 1–3 mg PA/kg bw/day for four-day up to two -week periods [28], and the World Health Organization (WHO) has established the daily intake limit of PAs of 10 μg/kg bw/day may cause HVOD in humans [29].

**Figure 6:**
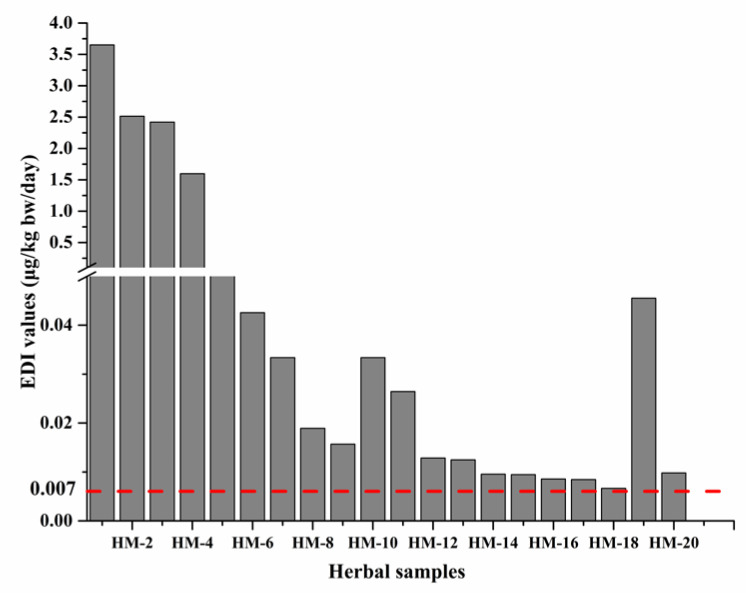
Estimated daily intake (EDI) values exceed the baseline for the consumption of PAs detected in Chinese herbal medicines. The red dashed line (- - -) in Figure represented the baseline 0.007 μg/kg bw/day.

Based on this, the 0.007 μg/kg bw/day was adopted as the tolerable maximum levels of exposure for PAs. Supplementary Material presented the EDI values of total PAs calculated for the consumption of positive herbal samples [30]. The values ranged from 4.28 × 10^−6^ to 3.652 μg/kg bw/day. As shown in Figure 6, the EDI values of all samples were well below the dose range of 1–3 mg PA/kg bw/day, these results indicated that the herbal consumers were not at risk for acute toxicity of PAs when consuming for short periods of 4 days up to 2 weeks. However, there were 20 herbal materials with EDI values above 0.007 μg/kg bw/day, which indicated that these medicines may have underlying toxicity. From the chart, the daily intake of PAs from sample HM-1 (3.652 μg/kg bw/day) was more than 521 times the baseline, followed by sample HM-2 (2.514 μg/kg bw/day), HM-3 (2.421 μg/kg bw/day), and HM-4 (1.597 μg/kg bw/day), at 359, 346, and 228 times, respectively.

#### 2.5.2. Chronic Exposure Scenario

**Figure 7:**
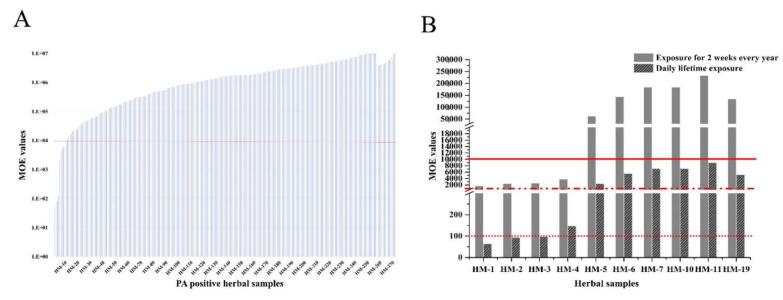
Margin of exposure (MOE) values obtained for the 271 PAs-positive herbal medicines (**A**), and top 10 PAs-positive herbal medicines with MOE values below 10,000 (**B**). Grey bars represent the MOE values calculated based on exposure for two weeks every year during a lifetime exposure, and the patterned bars represent daily lifetime exposure. The red solid line represents the MOE value of 10,000 (both for **A** and **B**) as a threshold for risk management action, and the red dash-dot line represents the MOE value of 1000 as a threshold for high priority, and the red dotted line (……) means the MOE value of 100 as top priority.

Chronic exposure risk assessment was conducted on 271 PA-positive herbal materials. The MOE value was calculated according to the Equation (2) assuming daily lifetime consumption and Equation (3) assuming two weeks of daily use every year during a lifetime, and the results are depicted in Table S8. EFSA classified the PAs materials into six priority bandings based on the MOE values [28], accordingly, the present study divided the herbal medicines into four grades: potentially toxic medicinal materials (MOE < 100); risk medicinal materials (100 ≤ MOE < 1000 ); low-risk medicinal materials (1000 ≤ MOE < 10,000); nonrisk medicinal materials (MOE ≥ 10,000). As shown in Figure 7A, for 10 out of 271 (3.6%) samples, the MOE values were below 10,000. There were three herbals with MOE values under 100, HM-1 (64.8), followed by HM-2 (94.2) and HM-3 (97.9), and just one sample with MOE value between 100 and 1000, HM-4 (148.3). Most respondents in China tended to take these herbal medicines only during a specified time period or when they had a worsened condition, therefore, the actual consumption periods of these herbals should be shorter, which was assumed to be two weeks/year. After correcting for shorter-than-life-time exposure, there were still four out of 271 (1.4%) herbal samples with MOE values below 10,000 (Figure 7B), namely HM-1 (1687.0), HM-2 (2450.7), HM-3 (2545.7), and HM-4 (3857.4), indicating that these herbals may pose a potential risk for human health.

## 3. Discussion

In the present study, we investigated the presence of PAs in 386 traditional Chinese herbal medicines recorded in the Chinese Pharmacopoeia (2020), with the aim to establish an efficient and sensitive analysis method and assess the potential health risk for consumers of these herbal medicines. 32 PAs standards can be well separated and maintain good peak shape by UPLC-MS/MS through the MRM mode, the sensitivity of all reference substances can reach 0.1 ng/mL, and some even up to 0.01 ng/mL.

Based on the above-established pretreatment method and UPLC-MS/MS analysis method, a total of 386 kinds of Chinese herbal medicines were detected and analyzed. PAs were detected in 271 out of 386 herbal medicine samples, accounting for more than 70%, and the PAs content were not less than 0.1 μg/kg. Among them, there were 10 samples with total PAs content more than 100 μg/kg, ranged from 121.9 to 25,567.4 μg/kg, and *A. euchroma* (25,567.4 μg/kg) was found to have the highest PA level. Senkirkine, intermedine and lycopsamine-*N*-oxide were the top three most frequently found PAs. Among these, the most widely distributed PAs was senkirkine, accounting for 69.7% (189/271) of the positive medicinal materials, followed by intermedine, which accounted for 45% (122/271). It was interesting to note that most of the high-content PAs samples are distributed in Compositae (38/271), Leguminosae (33/271), Ranunculaceae (11/271) and Labiatae (9/271); this finding is in general agreement with previous reports [5,6].

Our study revealed that there was a very wide variation in the EDI of PAs in different herbal medicines. This is due to the difference in their total PAs levels, as well as a wide range in the recommended daily maximum doses of the herbals recorded in the Chinese Pharmacopoeia (2020), ranging from 1.2 to 60 g per day. The highest daily intake of PAs was *A. euchroma* (255.6 μg/day), after calculation based on the EDI of 3.652 μg/kg bw/day and a body weight of 70 kg for adult [31]. There are 20 medicinal materials with EDI exceeded the limit 0.007 μg/kg bw/day, set by the EMA combined with the COT and BfR. The EDI of PAs from *A. euchroma* (3.652 μg/kg bw/day) was more than 521 times the baseline, which contained the highest PAs content, followed by *Tussilago farfara* (*T. farfara*, 2.514 μg/kg bw/day), *Eupatorium fortunei* (*E. fortune*, 2.421 μg/kg bw/day), and *Eupatorium lindleyanum* (*E. lindleyanum*, 1.597 μg/kg bw/day), at 359, 346, and 228 times, respectively.

When considering chronic exposure, the risk assessment was conducted on 271 PA-positive herbal medicines, and 10 out of 271 (3.6%) samples were below 10,000, and the MOE values for sample *A. euchroma* (64.8), *T. farfara* (94.2), and *E. fortunei* (97.9) were even lower than 100, indicating there should be a priority for risk management upon daily lifetime exposure. However, people tend to use these products for a short-term treatment. Hence, here we adopted Haber’s rule to evaluate the risk for shorter-than-lifetime exposure (two weeks every year during a lifetime) and correspondingly the MOE values were 26 times higher than the lifetime daily exposure MOE values. Accordingly, there were still four out of the 271 (1.48%) herbal samples with MOE values < 10,000, namely *A. euchroma* (1687.0), *T. farfara* (2450.7), *E. fortunei* (2545.7), and *E. lindleyanum* (3857.4), indicating that these herbals may have underlying toxicity, and the strict regulatory and quality control should be carried out to reduce the related health risk for consumers.

*A. euchroma* is one of the most used herbs to treat wounds and inflammation in many countries. There are at least 15 kinds of prescription preparations consisting of *A. euchroma* recorded in Chinese Pharmacopoeia (2020), e.g. Xiaoerfeireping Capsules. In this study, seven PAs were detected in *A. euchroma*, including europine, heliotrine, lycopsamine, echimidine, intermedine, echimidine-*N*-oxide, and intermedine-*N*-oxide, this finding is in line with the previous report [32]. As the commonly used herbs with the highest detected total PAs content (25,567.4 μg/kg), the potential hepatotoxicity of *A. euchroma* should be taken seriously.

*T. farfara* has a long history of medicinal use. It is listed as an “intermediate” medicine in China’s oldest Chinese medicine book “Shen Nong’s Bencao” (Han Dynasty, 25–220 AD). It is also recorded in “Compendium of Materia Medica” (Ming Dynasty, 1368–1644 AD), which described *T. farfara* as the drugs for treatment of chronic cough and phlegm syndrome with blood. Moreover, herbal preparations containing *T. farfara*, such as Ju Hong Tablets/Pills/Granula/Capsule, were frequently used to cure cough, asthma, and chronic bronchitis in China [33]. In this study, five PAs were detected in *T. farfara* with a total content of 17600.1 μg/kg, however, the Chinese Pharmacopoeia (2020) recommended daily maximum consumption dosage is 10 g, EDI value was more than 358 times the baseline, and such a large dose presents a huge safety risk.

*Gynura japonica* (*G. japonica*) has good effects of dispersing blood stasis, hemostasis and reducing swelling. Our present study revealed that six PAs were detected in *G. japonica*, with a total content of 531.0 μg/kg, and the Chinese Pharmacopoeia (2020) recommended daily maximum consumption dosage is 6 g, thus the obtained PAs daily maximum consumption is 3.1862 μg. *G. japonica* is contained in several commonly used clinical medicines, such as Sanqi Tablets. In fact, 15 patients poisoning cases were reported after taking these PA-containing *G. japonica* herbal products for five days up to two years, leading to HVOD, even death [34,35].

The WHO concluded that daily intake of PAs exceeding 10 µg/kg (bw) would lead to HVOD. Germany and the Netherlands has established maximum daily intake of PAs, which are 1 μg/day and 0.1 μg/day, respectively. So far, it has been stipulated that the medicinal materials shall not contain adonifoline (one kind of PAs) or its content shall not exceed 0.004%, in “Senecio” of Chinese Pharmacopoeia (2020). However, most of the traditional Chinese medicine lack the limitation of PAs, even less research has been done on herbals that have both medicinal and dietary uses, which greatly increase the exposure risk. Since several PAs poisoning cases were reported, we strongly suggest to strengthen the quality control of PAs in Chinese Pharmacopoeia.

However, it should be noted that, in the risk assessment of PAs, a worst-case approach is adopted, which assumes that the toxic potency of all PAs is similar to the highly toxic PA riddelliine [28]. In other words, this may result in an over-estimation of the risk assessment, because the existing in vivo and in vitro data [33,36] proposed that several PAs showed less toxic than riddelliine. The study [27] defined interim relative potency (iREP) factors for PAs, based on the available in vitro data of the genotoxicity potency in *Drosophila*, the different cytotoxicity in chicken hepatocellular carcinoma (CLR-2118) cells and in vivo data of acute toxicity in rodents. iREP describes the relative toxicity of each congener compared to the most toxic congener, the latter has a relative toxic efficacy (REP) coefficient of 1.0, which describes its toxic as 100%. For PAs, factors 1.0 is applied for cyclic diesters and heliotridine-type (7*S*) open diesters, 0.3 for heliotridine-type (7*S*) monoesters, 0.1 for retronecine-type (7*R*) open diesters, 0.01 for retronecine-type (7*R*) monoesters [27]. For PANOs, such as senecionine-*N*-oxide, retrorsine-*N*-oxide and lasiocarpine-*N*-oxide, iREP were equal to the corresponding free base PA. The recent study [37] based on the results of γH2AX analysis for 37 PAs in HepaRG human liver cells, showing that open chain diester and cyclic diester PAs have highest efficacy. 

Besides, we evaluated the MOE values according to the occurrence data obtained from comminuted herbal medicines extraction. However, a recent study showed that the PAs extraction efficiency from the comminuted tea leaves were 1.1–4.1 times higher than the intact form [38]. In view of the real life scenario that patients usually take these herbal medicines by hot water in partially intact form, the crushed form extraction may represent the worst case and does not show the real life consumption situation. As a result, our obtained PAs total content may higher than actual situation, and thus affecting the corresponding MOE values. Moreover, taking iREP factors into consideration in our further study may avoid overestimation of the risk assessment and promote more appropriate management measures for these traditional herbal medicines. 

## 4. Materials and Methods

### 4.1. Chemicals and Reagents

Thirty-four PAs analytical standards were all sourced from Phytolab (Vestenbergsgreuth, Germany), the structures of these compounds are shown in Figure S1 (Supplementary Material). Formic acid (HPLC grade) was obtained from DIKMA Technologies Inc. (Lake Forest, CA, USA) and ammonium bicarbonate (99% purity, HPLC grade) from MREDA Technologies Inc. (Beijing, China). Both ammonium hydroxide in water (NH_4_OH, 25%) and sulfuric acid (H_2_SO_4_, 98%) were purchased from Beijing Chemical Works (Beijing, China). Methanol (MeOH, UPLC/MS grade) was purchased from Thermo Fisher Scientific (Shanghai, China) and water from Wahaha Company (Hangzhou, China). Stock solutions of the 34 available PAs standards were prepared in acetonitrile (100 μg/mL) and stored at –20 °C. The standard working solution (1 µg/mL) consisted of mixed respective PAs reference standards in MeOH (HPLC grade) and stored at 4 °C.

### 4.2. Collection and Preparation of Samples

A total of 386 herbal medicines (100 g to 500 g each) were mainly purchased from three medicine markets and pharmacies of China (Anhui Bozhou Medicinal Materials Market, Hebei Baicao Kangshen Pharmaceutical Co., Ltd. (Hengshui City, China), and Beijing Tongrentang Pharmacy store). They were identified by Dr. Yulin Lin of Institute of Medicinal Plant Development, Chinese Academy of Medical Sciences and Peking Union Medical College (Beijing, China). Voucher specimens were deposited at the Institute of Medicinal Plant Development, Chinese Academy of Medical Sciences and Peking Union Medical College (Beijing, China). A summary of the source details is given in Table S8.

### 4.3. PAs Extraction

All these 386 types of herbal samples were smashed by using a high-speed mixer and the obtained powder was separately sieved through 60 mesh (0.3 mm). 2.0000 ± 0.0005 g portion of each homogeneous sample was accurately weighed and dissolved with 40 mL of MeOH, and then extracted by ultrasonic for 30 min at room temperature. All extractions were centrifuged at 6000 rpm (4430× *g*) for 10 min. The obtained supernatant was transferred to a new beaker (50 mL) and concentrated up to dryness, then reconstituted in 10 mL of 0.05 M sulfuric acid before the SPE procedure. 

The types of cartridges, sample loading amount, and eluents used during the extraction procedures can highly affect the recoveries of PAs from samples. In our study, five different kinds of SPE cartridges (500 mg/6 mL) were investigated in the form of recoveries by adding 1 mL of 100 ng/mL blank mixed acid standard solution to each of them, and the results are shown in Figure 3 (detailed recoveries are listed in the Supplementary Materials, Table S6). Compared to the widely fluctuated recoveries with Cleanert SCX and C8/SCX, Cleanert PCX had a more stable and higher recoveries ranged from 68.42% to 102.08% among the 34 PAs standards. Overall, Cleanert PCX was found to be the most appropriate clean-up cartridge. Hence, Cleanert PCX cartridges were used in the following LC-MS/MS analysis.

The PCX-SPE cartridges (500 mg, 6 mL) were preconditioned with 5 mL of MeOH and 5 mL of 0.05 M sulfuric acid, respectively. 10 mL sample was loaded and then washed with 5 mL of 0.05 M H_2_SO_4_ and 10 mL of MeOH. After that, the target PAs compounds were eluted with 10 mL of NH_4_OH/MeOH solution (1:3, *v*/*v*), which should be freshly prepared per working day. The obtained solutions were dried at 50 ℃ under nitrogen. The dried residues were reconstituted with 2 mL of MeOH/water (50:50, *v*/*v*) and then directly filtered into an amber LC vial (2 mL) using a syringe filter (0.22 µm).

### 4.4. LC-MS/MS Analysis

All herbal samples were separated and analyzed using an Agilent 6470 triple quadrupole mass spectrometer with Agilent Jet Stream technology in ESI positive ionization mode and an Agilent Infinity II 1290 UPLC system (Agilent Technologies, Palo Alto, CA, USA). The analytes were separated on a Zorbax Eclipse Plus C18 column (3.0 mm × 150 mm, 1.8 µm, Agilent Technologies) (more details see Table S1), and the column temperature was maintained at 40 °C (±0.8 °C). The mobile phase consisted of solvent A (water) and solvent B (MeOH), both mixture with 0.05% formic acid and 2.5 mM ammonium formate (0.5 min 95%A/5%B, 1.0 min 80%A/20%B, 11.0 min 63%A/37%B, 13.0 min 5%A/95%B, 15.5 min 5%A/95%B, 16.0 min 95%A/5%B), the flow rate is 0.4 mL/min. 2 μL of each sample extract was injected. The configuration and parameters are summarized in Tables S2 and S3.

Matrix effects often cause significant interferences and influences during the analysis process. A major problem of this approach is that the matrixes of those 386 Chinese herbal medicines are complicated and the PA/ PANO-free materials are hardly available in some matrices, especially those for herbals. In this study, *Perilla frutescens*, *Rhodiola rosea* L., *Syringa oblata* Lindl, and *Chaenomeles sinensis* were adopted as blank matrix samples, and mixed standards was added to these blank matrix samples. The matrix effect was calculated by comparison of the slopes of the calibration curve prepared by spiking blank herbal samples and calibration curve in solvent according to formula: ME = (slope_matrix_/slope_solvent_ − 1) ×100% [39]. According to the study [40,41], the matrix effects were considered to be soft if the values was in the range of 80–120%, the values above 120% indicated matrix effect was enhanced, while under 80% indicated matrix effect was suppressed. Consequently, as exhibited in Table 1, only Retronecine with ME under 50% showed suppressed effect, other components were all found to meet the requirements (80~120%).

Besides, validation of the analytical method was performed according to the Commission Decision No.2002/657/EC. The following parameters were determined: linearity, precision, recovery, LOD, and LOQ. Linearity of this method was studied using seven different concentration levels: the range from 0.1 ng/mL to 10 ng/mL for low-level compounds and the range from 10 ng/mL to 500 ng/mL for the high level. The recoveries of PAs were studied by analyzing triplicate samples spiked with mixed standards at three concentration levels of 1.0, 10.0 and 100.0 ng/mL. The precision of the method was evaluated by performing tests on six replicated injections of the same spiked samples. The intra-day and interday repeatability were also estimated by analyzing spiked samples in a single day and for three different consecutive days, respectively. The LOD and LOQ were determined at the signal-to-noise (S/N) ratios of 3 and 10, respectively.

### 4.5. Estimated Daily Intake (EDI) of PAs Resulting from the Consumption of Herbal Medicines

EDI was calculated according to Equation (1)

EDI = (C × M)/(BW × 1000)(1)

The EDI values are expressed in μg/kg bw/day. C is the total content of PAs detected in the PA-positive samples (271/386) by LC-MS/MS, expressed in μg/kg. M is the Chinese Pharmacopoeia (2020) recommended maximum daily consumption dosage of these herbal samples, expressed in g. The factor 1000 is used to convert M in g to kg. BW is body weight of 70 kg proposed by the European Food Safety Authority (EFSA) [31].

To evaluate the noncancer effects of PAs, the British COT came to the conclusion that doses of PAs below 0.007 μg/kg bw/day, would unlikely be of concern [42]. Accordingly, the BfR identified that, for 1,2-unsaturated PAs, a daily intake of 0.007 μg/kg (0.42 μg/60 kg adult) should not be exceeded [43]. Also the Committee on Herbal Medicinal Products (HMPC) of the EMA concluded that the short-time (maximum 14 days) daily intake of 0.35 µg (50 kg adult) toxic unsaturated PAs/day from herbal medicinal products might be acceptable [44]. Based on these, the tolerable levels of exposure for PAs should not exceed 0.007 µg/kg bw/day, and 0.49 µg/day was adopted as the baseline value of risk assessment for adults with a body weight of 70 kg.

The risk assessment of PAs was calculated using the recommended maximum daily intakes in the Chinese Pharmacopoeia (2020), and the ones exceeding the baseline value are shown in Table 2.

### 4.6. Calculation of MOE

EFSA proposed that MOE approach can be applied for the risk assessment of substances that have both genotoxic and carcinogenic properties [45], as shown in Equation (2), the MOE value was calculated by dividing the reference point BMDL_10_ by the EDIz [30].

MOE = BMDL_10_/EDI (2)

where the MOE is a ratio, the BMDL_10_ value used was 237 μg/kg bw/day originated from a chronic exposure experiment with riddelliine in rats [24]. MOE values below 10,000 indicate that there might be a potential concern for human health.

### 4.7. Actual Life Exposure of Chinese Herbal Medicines

The MOE values are based on chronic lifetime exposure. While most respondents in China tended to take these herbal medicines only during a specified time period or when they had a worsened condition, therefore, the actual consumption periods of these herbals should be shorter, which is assumed to be two weeks/year.

Here we adopted Haber’s rule [30,46] to correct the EDI and thus the MOE approach, and the MOE (two weeks/year during a lifetime) of PAs can be expressed as follows [38]:

MOE (two weeks/year during a lifetime) = MOE × 26(3)

where the MOE on the right side of the equation is acquired by Equation (2).

## 5. Conclusions

In conclusion, the established efficient and sensitive UPLC-MS/MS method could be used for the detection of toxic PAs in herbs. *A. euchroma*, *T. farfara*, *E. fortunei*, and *E. lindleyanum* may pose a potential risk for human health, when consumed for two weeks a year at the dosage of Chinese Pharmacopoeia (2020) recommended maximum daily consumption. This study provides a meaningful reference for the rational use of Chinese herbal medicines, and the results of the risk assessment highlight the urgency for regulatory actions, with the aim to reduce the level of PAs that occur in these products. In addition, it is necessary to carry out more in-depth investigations to ensure the safe use of these Chinese herbal medicines.

## Figures and Tables

**Table 1:** Matrix effects of PAs/ pyrrolizidine alkaloid *N*-oxides (PANOs) in *P. frutescens*, *R. rosea*, *S. oblata*, *C. sinensis.*

PAs		*P. frutescens*		Average		*R. rosea*		Average		*S. oblata*		Average		*C. sinensis*		Average
Abbr.	50 μg/kg	100 μg/kg	200 μg/kg		50 μg/kg	100 g/kg	200 μg/kg		50 μg/kg	100 μg/kg	200 μg/kg		50 μg/kg	100 μg/kg	200 μg/kg	
Ret	52.96	50.23	44.33	**49.17**	34.05	31.48	28.58	**31.37**	33.80	32.45	28.92	**31.73**	28.29	25.93	22.58	**25.60**
Mc	87.51	81.26	76.29	**81.69**	93.52	83.99	81.09	**86.20**	80.36	77.07	73.45	**76.96**	91.93	87.35	83.57	**87.62**
Er	92.22	82.64	79.19	**84.68**	92.24	83.75	80.78	**85.59**	82.58	77.49	75.82	**78.63**	95.34	89.42	86.64	**90.47**
McNO	93.11	87.48	84.56	**88.39**	99.22	92.92	93.90	**95.35**	91.41	88.53	85.60	**88.51**	95.78	92.95	90.91	**93.21**
ErNO	93.30	88.68	81.93	**87.97**	99.11	94.69	91.74	**95.18**	92.86	88.15	84.76	**88.59**	97.35	93.44	91.38	**94.06**
Jb	95.29	89.09	84.03	**89.47**	95.34	87.42	83.72	**88.83**	87.94	82.76	80.34	**83.68**	96.02	90.20	87.06	**91.09**
Eu	116.50	109.30	102.67	**109.49**	106.92	98.97	98.63	**101.51**	123.26	114.61	111.40	**116.42**	115.77	108.56	104.45	**109.59**
Im	104.19	96.34	91.69	**97.41**	100.51	91.80	87.10	**93.14**	101.71	95.22	94.75	**97.23**	101.15	94.47	93.20	**96.27**
Ly	112.08	99.29	91.32	**100.90**	118.46	102.36	97.05	**105.96**	119.29	108.72	107.15	**111.72**	117.58	105.60	104.14	**109.11**
JbNO	92.05	86.68	83.15	**87.29**	95.84	91.72	93.77	**93.77**	90.24	87.77	86.67	**88.23**	99.58	93.67	93.10	**95.45**
EuNO	113.80	106.74	102.79	**107.77**	112.71	103.62	106.20	**107.51**	111.69	108.06	106.05	**108.60**	111.70	103.73	105.91	**107.11**
ImNO	136.31	125.89	120.85	**127.68**	127.33	115.07	119.21	**120.54**	133.24	122.01	123.50	**126.25**	129.96	116.92	120.13	**122.34**
LyNO	114.13	106.84	103.62	**108.20**	111.30	105.04	108.91	**108.41**	112.67	106.88	106.47	**108.67**	114.17	106.61	107.32	**109.37**
Re	88.24	85.83	81.80	**85.29**	98.16	92.80	87.90	**92.95**	96.13	94.37	91.86	**94.12**	99.43	96.15	93.41	**96.33**
ReNO	97.58	92.65	88.85	**93.03**	101.48	96.09	98.72	**98.76**	101.12	93.88	94.61	**96.53**	103.04	97.81	97.75	**99.53**
Td	98.47	93.47	87.79	**93.25**	99.88	92.35	92.91	**95.05**	96.87	93.51	92.58	**94.32**	98.56	94.97	94.82	**96.12**
Sp	100.76	94.30	88.73	**94.60**	101.32	91.52	87.85	**93.57**	102.39	93.53	94.67	**96.86**	103.53	95.24	93.72	**97.50**
7-Im	108.18	101.53	94.91	**101.54**	107.08	98.40	96.02	**100.50**	107.56	104.56	100.25	**104.12**	103.72	100.51	96.29	**100.17**
He	109.93	102.09	97.39	**103.14**	107.59	98.34	96.99	**100.97**	108.75	103.93	100.47	**104.39**	106.52	101.40	98.85	**102.26**
7-Ly	111.21	100.62	96.59	**102.81**	107.41	96.70	96.79	**100.30**	111.66	101.03	98.92	**103.87**	107.92	99.05	97.60	**101.52**
SpNO	99.31	95.89	91.65	**95.62**	100.17	94.36	98.88	**97.80**	100.08	95.67	95.34	**97.03**	102.86	97.71	99.56	**100.04**
7ImNO	80.81	77.37	75.52	**77.90**	89.81	86.90	88.52	**88.41**	83.31	81.43	79.87	**81.54**	88.80	87.43	85.12	**87.12**
HeNO	105.60	97.82	95.78	**99.74**	106.34	96.47	102.04	**101.62**	104.15	99.02	99.01	**100.73**	104.00	98.86	98.52	**100.46**
Sv	100.08	93.29	87.62	**93.66**	99.35	89.16	85.48	**91.33**	99.25	93.91	92.12	**95.09**	99.89	91.94	91.49	**94.44**
Sn	101.29	96.03	90.98	**96.10**	101.42	92.35	87.74	**93.84**	100.07	94.97	93.77	**96.27**	100.19	95.83	95.42	**97.15**
SvNO	104.14	97.65	94.73	**98.84**	105.24	99.60	102.36	**102.40**	104.85	99.38	96.48	**100.24**	107.33	99.20	99.17	**101.90**
SnNO	103.66	96.53	94.35	**98.18**	105.18	99.56	101.74	**102.16**	103.00	95.50	95.41	**97.97**	101.84	99.27	99.26	**100.12**
Em	107.55	101.47	99.18	**102.73**	105.32	99.30	98.47	**101.03**	106.05	102.22	100.38	**102.88**	105.90	100.02	98.84	**101.59**
EmNO	108.87	107.19	113.70	**109.92**	111.86	113.25	129.58	**118.23**	112.14	111.47	116.28	**113.29**	113.34	113.89	125.30	**117.51**
Sk	105.56	100.38	95.60	**100.51**	105.89	100.33	104.05	**103.42**	105.62	101.63	99.82	**102.36**	106.31	101.57	101.08	**102.99**
Lc	92.85	88.45	90.21	**90.50**	94.69	89.25	93.62	**92.52**	89.74	87.28	91.01	**89.34**	97.76	93.19	95.74	**95.56**
LcNO	90.74	85.54	81.06	**85.78**	94.34	88.08	90.09	**90.84**	85.59	81.88	81.63	**83.03**	96.76	90.92	92.21	**93.30**
Ret	52.96	50.23	44.33	**49.17**	34.05	31.48	28.58	**31.37**	33.80	32.45	28.92	**31.73**	28.29	25.93	22.58	**25.60**
Mc	87.51	81.26	76.29	**81.69**	93.52	83.99	81.09	**86.20**	80.36	77.07	73.45	**76.96**	91.93	87.35	83.57	**87.62**

**Table 2:** The PA content detected in herbal medicines over the baseline, and the corresponding EDI and MOE values.

Sample ID	Herbal Samples	Numbers of PAs Detected and Top Three	Total PAs Content (μg/kg)	Recommended Maximum Daily Use (g)	PAs Maximum Daily Consumption (g)	EDI (μg/kg bw/ Day)	MOE Values (Life Long Time)	MOE Values (Shorter Exposure)
HM-1	*Arnebia euchroma*	7 (LyNO, ImNO, EmNO)	25567.4	10	255.6	3.652	64.8	1687.0
HM-2	*Tussilago farfara*	10 (Sk, SnNO, Sn)	17600.2	10	176.0	2.514	94.2	2450.7
HM-3	*Eupatorium fortunei*	5 (Im, Ly, LyNO)	16943.6	10	169.4	2.420	97.9	2545.7
HM-4	*Eupatorium lindleyanum*	5 (LyNO, Im, Ly)	1863.6	60	111.8	1.597	148.3	3857.4
HM-5	*Senecio scandens*	12 (Sk, SnNO, SpNO)	229.0	30	6.8	0.098	2414.5	62,777.8
HM-6	*Laggera pterodonta*	9 (Im, ImNO, JbNO)	198.5	15	2.9	0.042	5569.5	144,808.1
HM-7	*Artemisia scoparia*	6 (Im, Ly, LyNO)	233.6	10	3.5	0.033	7100.9	184,625.2
HM-8	*Cassia angustifolia*	9 (Mc, McNO, Td)	220.6	6	1.3	0.018	12,528.8	325,751.0
HM-9	*Euphorbia hirta*	6 (Im, ImNO, LyNO)	121.9	9	1.0	0.015	15,121.6	393,163.7
HM-10	*Gynura japonica*	6 (SnNO, SpNO, Sk)	531.0	6	3.1	0.045	5206.7	135,375.7
HM-11	*Scutellaria barbata*	5 (LyNO, Ly, ImNO)	61.7	30	1.8	0.026	8958.3	232,917.5
HM-12	*Euphorbia humifusa*	6 (LyNO, Ly, ImNO)	45.0	20	0.9	0.012	18,421.0	478,947.3
HM-13	*Picria fel-terrae*	6 (LyNO, Ly, Im)	58.2	15	0.8	0.012	18,983.8	493,580.5
HM-14	*Cirsium japonicum*	7 (Im, Sk, LyNO)	44.6	15	0.6	0.009	24,798.2	644,753.3
HM-15	*Abrus cantoniensis*	6 (LyNO, Ly, ImNO)	22.1	30	0.6	0.009	25,067.9	651,767.9
HM-16	*Equisetum hyemale*	6 (LyNO, Ly, ImNO)	66.6	9	0.5	0.008	27,677.6	719,619.6
HM-17	*Apocynum venetum*	5(LyNO, Ly, ImNO)	49.3	12	0.5	0.008	28,071.0	729,847.7
HM-18	*Siphonostegia chinensis*	13 (Im, ImNO, LyNO)	51.8	9	0.4	0.006	35,530.7	923,798.5
HM-19	*Achillea alpina*	4 (Sk, Im, Ly)	51.9	45	2.3	0.033	7103.4	184,688.5
HM-20	*Phryma leptostachya*	6 (LyNO, Ly, ImNO)	45.7	15	0.6	0.009	24,179.6	628,671.4

## Data Availability

Not applicable.

## Supplementary Materials

The following are available online. Figure S1: Chemical structures of 34 PAs, Table S1: MS/MS compound information and retention time (RT) of the PAs analytes, Table S2: UPLC system Configuration and parameters, Table S3: UPLC triple quadrupole mass spectrometer configuration and parameters, Table S4: The concentrations of detected PAs in A. capillaris with different extractant solvents (n = 3, μg/kg), Table S5: The concentrations of detected PAs in S.scandens with different extractant solvents (n = 3, μg/kg), Table S6: The detailed recoveries of each PA with different SPE cartridges, Table S7: LOD, LOQ, recoveries, intra-day and inter-day repeatability obtained by UPLC-MS/MS method, Table S8: The PA content detected in all 386 herbal medicines, and the corresponding EDI and MOE values.
