# Supplementary material for: Determination of Toxic Pyrrolizidine Alkaloids in Traditional Chinese Herbal Medicines by UPLC-MS/MS and Accompanying Risk Assessment for Human Health
Source: Molecules. 2021 Mar 16;26(6):1648. doi: 10.3390/molecules26061648 (PMC8000276; doi:10.3390/molecules26061648)
Supplement: Supplementary file 1 [file molecules-26-01648-s001.pdf]

Supplementary Material

Determination of toxic pyrrolizidine alkaloids in traditional Chinese herbal medicines by UPLC-MS/MS and accompanying risk assessment for human health

Junchi Wang [1,†], Meng Zhang [1,†], Lihua Chen [1,2], Yue Qiao [1], Siqi Ma [1], Dian Sun [1], Jianyong Si [1,*] and Yonghong Liao [1,*]

[1] The Key Laboratory of Bioactive Substances and Resources Utilization of Chinese Herbal Medicine, Ministry of Education, Institute of Medicinal Plant Development, Chinese Academy of Medical Sciences & Peking Union Medical College, Beijing 100193, China.; jcwang@implad.ac.cn (J.W.); mandy6378@126.com (M.Z.); lihuachen0706@163.com (L.C.); MOON100107qy@163.com (Y.Q.); siqimazry@163.com (S.M.); dasun@implad.ac.cn (D.S.)

[2] State Key Laboratory of Natural and Biomimetic Drugs, School of Pharmaceutical Sciences, Peking University, Beijing 100191, China

* Correspondence: jysi@implad.ac.cn (J. S.); yhliao@implad.ac.cn (Y. L.); Tel.: +86-10-5783-3299 (J. S.); +86-10-5783-3268 (Y. L.)

† These authors contributed equally to this work.

Contents

Figure S1. Chemical structures of 34 PAs.

Table S1. MS/MS compound information and retention time (RT) of the PAs analytes

Table S2. UPLC system Configuration and parameters

Table S3. UPLC triple quadrupole mass spectrometer configuration and parameters

Table S4. The concentrations of detected PAs in *A. capillaris* with different extractant solvents (n = 3, μg/kg)

Table S5. The concentrations of detected PAs in *S.scandens* with different extractant solvents (n = 3, μg/kg)

Table S6. The detailed recoveries of each PA with different SPE cartridges

Table S7. LOD, LOQ, recoveries, intra-day and inter-day repeatability obtained by UPLC-MS/MS method.

Table S8. The PA content detected in all 386 herbal medicines, and the corresponding EDI and MOE values

retronecine-type:

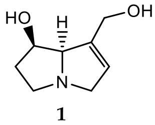

1

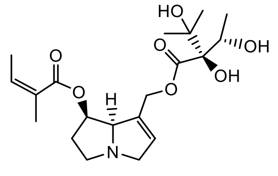

2

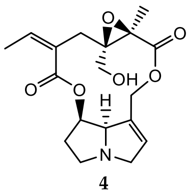

4

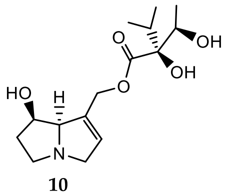

10

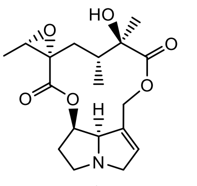

12

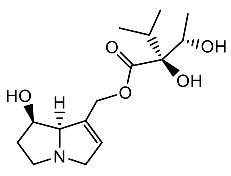

16

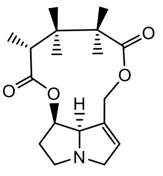

18

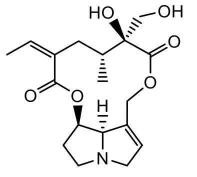

20

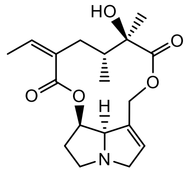

22

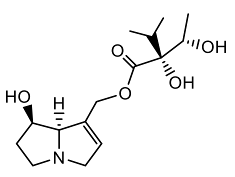

28

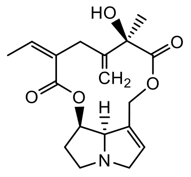

24

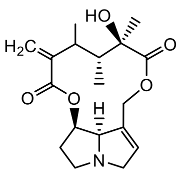

26

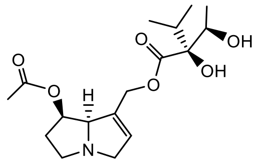

30

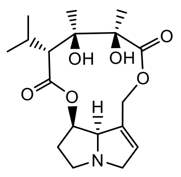

33

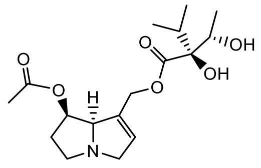

34

retronecine N-oxide-type:

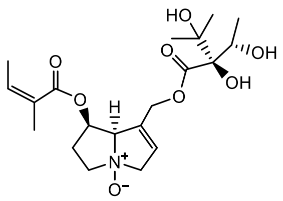

3

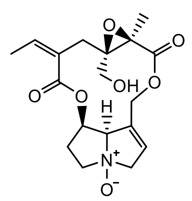

5

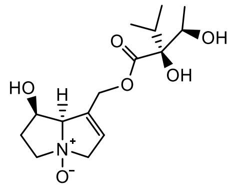

11

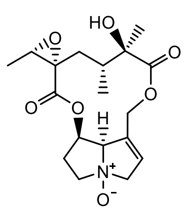

13

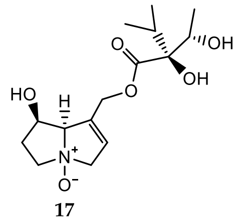

17

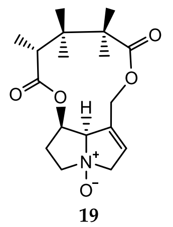

19

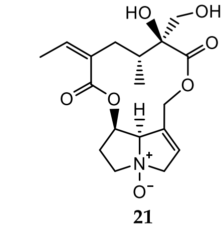

21

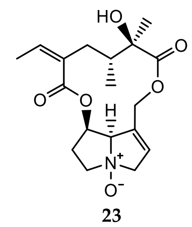

23

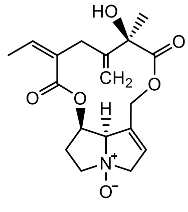

25

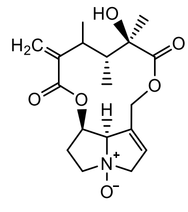

27

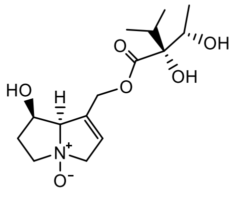

29

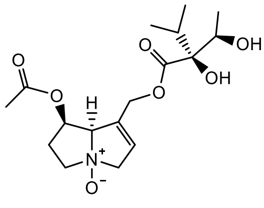

31

heliotridine-type: **Otonecine-type :**

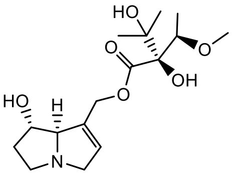

6

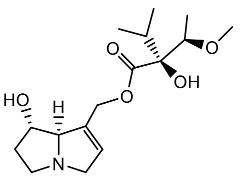

8

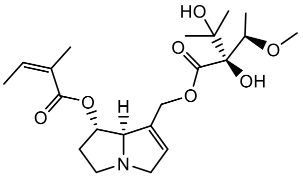

14

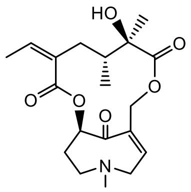

32

heliotridine N-oxide-type:

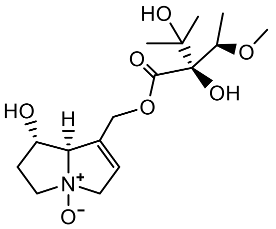

7

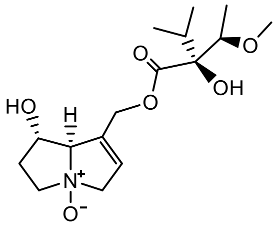

9

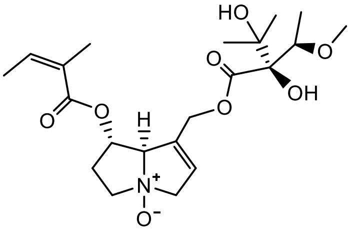

15

Figure S1. Chemical structures of 34 PAs.

retronecine-type : **1**: retronecine, **2**: echimidine, **4**: erucifoline, **10**: intermedine, **12**: jacobine, **16**: lycopsamine, **18**: monocrotaline, **20**: retrorsine, **22**: senecionine, **24**: seneciphylline, **26**: senecivernine, **28**: indicine, **30**: 7-acetylintermedine, **33**: trichodesmine, **34**: 7-acetyllycopsamine; **retronecine N-oxide-type: 3**: echimidine N-oxide, **5**: erucifoline N-oxide, **11**: intermedine N-oxide, **13**: jacobine N-oxide, **17**: lycopsamine N-oxide, **19**: monocrotaline N-oxide, **21**: retrorsine N-oxide, **23**: senecionine N-oxide, **25**: seneciphylline N-oxide, **27**: senecivernine N-oxide, **29**: indicine N-oxide, **31**: 7-acetylintermedine N-oxide; **heliotridine-type: 6**: europine, **8**: heliotrine, **14**: lasiocarpine; **heliotridine N-oxide-type: 7**: europine N-oxide, **9**: heliotrine N-oxide, **15**: lasiocarpine N-oxide; **otonecine-type: 32**: senkirkine.

Table S1. MS/MS compound information and retention time (RT) of the PAs analytes

Num.	Compound Name	Abbr.	RT (min)	Precursor Ion (m/z)	Product Ion (m/z)	Fragmentor (V)	CE (V)
1	retronecine	Ret	1.66	156.2	112 [a], 108.1	106	21
2	echimidine	Em	11.41	398.2	220, 120 [a]	151	17
3	echimidine N-oxide	EmNO	11.59	414.2	352.1, 254.1 [a]	152	33
4	erucifoline	Er	3.66	350.2	138, 120 [a]	167	33, 29
5	erucifoline N-oxide	ErNO	4.22	366.2	136 [a], 120.1	159	37
6	europine	Eu	4.54	330.2	138 [a], 156	131	21, 33
7	europine N-oxide	EuNO	5.07	346.2	172 [a], 111	144	33, 53
8	heliotrine	He	7.19	314.2	156, 138 [a]	149	33, 21
9	heliotrine N-oxide	HeNO	8.16	330.2	172, 138 [a]	149	29
10	intermedine	Im	4.67	300.2	156, 138 [a]	144	33, 21
11	intermedine N-oxide	ImNO	5.51	316.2	172 [a], 138	152	33
12	jacobine	Jb	4.43	352.2	155, 120 [a]	167	33
13	jacobine N-oxide	JbNO	4.94	368.2	296.1 [a], 120	157	25, 41
14	lasiocarpine	Lc	13.61	412.2	336.2, 120 [a]	134	17, 33
15	lasiocarpine N-oxide	LcNO	13.89	428.2	254.1 [a], 136.1	162	33
16	lycopsamine	Ly	4.85	300.2	156, 138 [a]	149	33, 21
17	lycopsamine N-oxide	LyNO	5.75	316.2	172 [a], 138	149	29
18	monocrotaline	Mc	3.46	326.2	237.2, 120 [a]	157	30, 37
19	monocrotaline N-oxide	McNO	4.21	342.2	137 [a], 118.1	167	33, 37
20	retrorsine	Re	6.10	352.2	138, 120.1 [a]	177	33
21	retrorsine N-oxide	ReNO	6.56	368.2	136, 118.1 [a]	172	41, 33
22	senecionine	Sn	8.89	336.2	120.1 [a], 138	185	33
23	senecionine N-oxide	SnNO	9.71	352.2	136, 118 [a]	167	37, 45
24	seneciphylline	Sp	6.86	334.2	138, 120 [a]	162	29
25	seneciphylline N-oxide	SpNO	7.64	350.2	136, 120.1 [a]	154	37
26	senecivernine	Sv	8.66	336.2	138.1, 120 [a]	177	33
27	senecivernine N-oxide	SvNO	9.35	352.2	136, 118.1 [a]	162	37
28	indicine	Ic	4.67	300.2	156.1, 138 [a]	141	33, 21
29	indicine N-oxide	IcNO	5.51	316.2	172 [a], 138	154	29
30	7-acetylintermedine	7-Im	7.18	342.2	180, 120 [a]	144	17, 29
31	7-acetylintermedine N-oxide	7-ImNO	7.65	358.2	214 [a], 137	157	29, 33
32	senkirkine	Sk	12.13	366.2	168 [a], 150	157	33, 29
33	trichodesmine	Td	6.52	354.2	222.1 [a], 120.1	162	33, 45
34	7-acetyllycopsamine	7-Ly	7.36	342.2	180, 120.1 [a]	146	17, 25

a: Ions for quantitative determination

Table S2. UPLC system Configuration and parameters

Configuration	Parameters		
Pump	Two Agilent 1290 Infinity II binary pumps (p/n G7120A)		
Multisampler/autosampler	Agilent 1290 Infinity II multisampler 108-vial well-plate trays (p/n G7167B)		
Column compartment	Agilent 1290 Infinity thermostatted column compartment with a 2-position 10 port valve (p/n G7116B)		
Analytical column	Agilent EclipsePlus C18, 3.0 mm × 150 mm, 1.8 µm ZORBAX LC column (p/n 959759-302)		
Column temperature	40°C		
Needle Wash Mode	Flush Port		
Injection volume	2.00 µL		
Mobile phase A	0.05% Formic acid and 2.5 mM/L Ammonium formate water		
Mobile phase B	0.05% Formic acid and 2.5 mM/L Ammonium formate in methanol		
Flow rate	0.400 mL/min		
Gradient	Time (min)	A (%)	B (%)
	0.50	95	5
	1.00	80	20
	11.00	63	37
	13.00	5	95
	15.50	5	95
	15.60	95	5
Stop time	19.00 min		

Table S3. UPLC triple quadrupole mass spectrometer configuration and parameters

**Configuration**	
Instrument	Agilent 6470 triple quadrupole mass spectrometer with Agilent Jet Stream
Ion mode	ESI + Agilent Jet Stream
Scan Type	MRM
Delta EMV	300 V
Ionization mode	Positive
Dwell time	5 ms
Cell accelerator voltage	3 V
**Source Parameters**	
Drying gas temperature	300°C
Drying gas flow	7 L/min
Nebulizer pressure	40 psi
Sheath gas Heater	325°C
Sheath gas flow	11 L/min
Capillary voltage	3500 V
Q1/Q2 resolution	Unit/Enh

Table S4. The concentrations of detected PAs in *A. capillaris* with different extractant solvents (n = 3, μg/kg)

Solvents	Methanol	0.05 M Acid methanol	0.05 M Sulfuric acid	0.05 M Acid ethanol	Ethanol
Total PAs	133.1	104.0	88.8	85.4	57.6
	127.2	101.1	94.7	88.1	50.5
	140.0	110.2	92.6	90.0	65.4
Mean	133.4	105.1	92.0	87.8	57.8
SD	6.41	4.64	2.99	2.31	7.45

Table S5. The concentrations of detected PAs in *S.scandens* with different extractant solvents (n = 3, μg/kg)

Solvents	Methanol	0.05 M Acid methanol	0.05 M Sulfuric acid	0.05 M Acid ethanol	Ethanol
Total PAs	146.2	127.3	82.4	80.0	63.4
	140.3	119.5	84.4	93.2	58.8
	150.7	103.2	89.7	81.3	52.6
Mean	145.7	116.7	85.5	84.8	58.3
SD	5.19	12.29	3.75	7.28	5.41

Table S6. The detailed recoveries of each PA with different SPE cartridges

No.	PCX [a]	C18 [b]	SCX [c]	C8/SCX [d]	X-C [e]	No.	PCX	C18	SCX	C8/SCX	X-C
1	75.38%	68.24%	107.46%	61.38%	76.89%	18	88.38%	77.05%	83.12%	88.79%	80.17%
2	79.41%	80.37%	84.99%	78.74%	92.89%	19	101.59%	54.40%	79.99%	64.66%	83.02%
3	91.38%	93.33%	73.38%	80.06%	89.81%	20	87.07%	76.64%	87.59%	71.32%	92.80%
4	71.82%	80.63%	58.14%	73.39%	69.63%	21	95.93%	92.52%	75.35%	86.44%	70.76%
5	87.15%	99.71%	48.29%	60.82%	79.91%	22	87.74%	77.44%	80.83%	93.37%	92.38%
6	97.25%	89.55%	91.41%	71.92%	64.50%	23	94.08%	94.42%	61.97%	75.08%	79.63%
7	95.74%	91.68%	88.87%	78.22%	84.72%	24	81.92%	78.45%	81.85%	87.88%	89.37%
8	92.67%	84.98%	91.15%	77.47%	86.62%	25	79.47%	88.94%	55.83%	73.25%	83.36%
9	93.88%	91.78%	85.49%	80.03%	89.48%	26	82.63%	76.95%	84.89%	88.32%	71.55%
10	91.45%	78.33%	93.52%	74.47%	77.45%	27	89.94%	89.14%	64.00%	81.20%	87.18%
11	96.50%	100.55%	82.27%	76.66%	99.71%	28	85.91%	77.58%	91.25%	74.47%	77.45%
12	80.18%	81.61%	83.69%	68.21%	66.15%	29	98.72%	98.81%	83.43%	76.66%	99.71%
13	80.67%	95.83%	63.02%	66.56%	83.66%	30	81.60%	80.03%	80.06%	75.49%	80.73%
14	89.46%	86.06%	88.41%	85.91%	80.41%	31	68.42%	109.95%	69.72%	71.87%	78.94%
15	97.82%	90.83%	87.93%	91.36%	77.02%	32	102.08%	86.41%	94.38%	82.21%	81.12%
16	85.91%	77.58%	91.25%	75.35%	76.72%	33	90.04%	74.12%	89.18%	60.20%	87.87%
17	98.72%	98.81%	83.43%	87.49%	91.73%	34	83.40%	80.27%	82.14%	71.16%	89.83%

a: Cleanert PCX b: Cleanert C18 c: Cleanert SCX d: Cleanert C8/SCX e: Strata X-C.

1: retronecine, **2:** echimidine, **3:** echimidine N-oxide, **4:** erucifoline, **5:** erucifoline N-oxide, **6:** europine, **7:** europine N-oxide, **8:** heliotrine, **9:** heliotrine N-oxide, **10:** intermedine, **11:** intermedine N-oxide, **12:** jacobine, **13:** jacobine N-oxide, **14:** lasiocarpine, **15:** lasiocarpine N-oxide, **16:** lycopsamine, **17:** lycopsamine N-oxide, **18:** monocrotaline, **19:** monocrotaline N-oxide, **20:** retrorsine, **21:** retrorsine N-oxide, **22:** senecionine, **23:** senecionine N-oxide, **24:** seneciphylline, **25:** seneciphylline N-oxide, **26:** senecivernine, **27:** senecivernine N-oxide, **28:** indicine, **29:** indicine N-oxide, **30:** 7-acetylintermedine, **31:** 7-acetylintermedine N-oxide, **32:** senkirkine, **33:** trichodesmine, **34:** 7-acetyllycopsamine.

Table S7. LOD, LOQ, recoveries, intra-day and inter-day repeatability obtained by UPLC-MS/MS method.

No.	PAs Abbr.	Regression equation	R^2	LOD μg/kg	LOQ μg/kg	Recoveries (%) Mean ± SD, (n = 3) 1 μg/kg	10 μg/kg	100 μg/kg	Intra-Day RSD%, n = 6	Inter-Day RSD%, n = 6
1	Ret	Y = 7993.54X - 2154.35	0.990	0.10	0.50	68.10 ± 0.35	75.38 ± 1.16	74.78 ± 2.12	4.54	3.32
2	Em	Y = 60260.15X - 1975.58	0.995	0.05	0.20	74.78 ± 0.18	79.41 ± 0.54	85.55 ± 1.16	0.94	5.04
3	EmNO	Y = 27531.43X - 2097.35	0.999	0.10	0.20	74.96 ± 0.21	91.38 ± 0.41	89.86 ± 0.78	1.51	7.64
4	Er	Y = 15310.77X - 872.51	0.999	0.02	0.20	67.84 ± 1.55	71.82 ± 2.31	74.57 ± 1.02	0.59	4.37
5	ErNO	Y = 7478.06X - 97.80	0.998	0.10	0.50	71.41 ± 1.24	87.15 ± 2.03	86.34 ± 1.43	1.78	3.27
6	Eu	Y = 109826.29X - 9271.26	0.999	0.01	0.10	73.33 ± 1.57	97.25 ± 1.14	91.47 ± 2.18	1.04	2.55
7	EuNO	Y = 48724.64X - 3244.91	0.999	0.02	0.10	82.25 ± 0.78	95.74 ± 2.24	97.40 ± 1.52	2.32	2.35
8	He	Y = 93885.61X - 9643.91	0.998	0.01	0.10	73.34 ± 0.35	92.67 ± 0.23	90.08 ± 0.80	0.65	2.81
9	HeNO	Y =10201.22X - 928.37	0.999	0.10	0.20	79.93 ± 0.46	93.88 ± 0.33	93.00 ± 2.25	1.86	3.76
10	Im	Y = 142989.86X - 14002.18	0.996	0.01	0.10	81.97 ± 1.34	91.45 ± 1.07	92.80 ± 1.11	0.79	4.72
11	ImNO	Y = 118910.82X - 5558.31	0.999	0.05	0.20	96.15 ± 1.47	96.50 ± 3.01	91.15 ± 2.38	6.88	4.04
12	Jb	Y = 11478.94X - 517.65	0.999	0.10	0.50	77.63 ± 0.42	80.18 ± 1.12	78.82 ± 3.46	1.73	4.72
13	JbNO	Y = 16685.77X - 532.96	0.999	0.10	0.50	71.64 ± 1.53	80.67 ± 2.45	81.99 ± 3.12	3.99	3.71
14	Lc	Y = 115049.54X - 3750.58	0.998	0.01	0.05	71.75 ± 0.57	89.46 ± 0.53	84.90 ± 5.12	1.89	4.48
15	LcNO	Y = 21287.08X - 861.07	0.999	0.05	0.20	73.70 ± 0.95	97.82 ± 2.58	91.51 ± 0.31	2.43	5.05
16	Ly	Y = 108555.30X - 11663.52	0.997	0.01	0.10	76.47 ± 0.07	85.91 ± 1.65	89.52 ± 1.26	3.32	4.45
17	LyNO	Y = 93065.10X - 4944.57	0.999	0.05	0.2	101.58 ± 1.43	98.71 ± 1.53	93.08 ± 2.03	2.81	3.23
18	Mc	Y = 25086.29X - 935.51	0.999	0.02	0.10	74.01 ± 1.14	88.38 ± 2.14	89.62 ± 1.68	0.38	4.35
19	McNO	Y = 8998.56X - 61.22	0.998	0.20	0.50	84.62 ± 0.50	101.59 ± 1.09	92.46 ± 1.34	2.30	2.86
20	Re	Y = 11752.29X - 48.81	0.998	0.05	0.50	78.97 ± 0.78	87.07 ± 0.55	88.80 ± 4.61	2.11	5.09
21	ReNO	Y = 4898.76X + 261.46	0.994	0.10	0.50	74.40 ± 0.62	95.93 ± 2.32	86.89 ± 6.07	2.42	6.87
22	Sn	Y = 18561.66X - 859.85	0.998	0.10	0.50	71.52 ± 0.85	87.74 ± 0.76	84.53 ± 7.43	1.24	1.10
23	SnNO	Y = 8959.16X - 333.55	0.999	0.10	0.50	73.22 ± 0.98	94.08 ± 1.04	89.74 ± 0.87	0.47	3.58
24	Sp	Y = 17075.87X - 1076.93	0.999	0.10	0.50	69.65 ± 1.34	81.92 ± 4.34	80.32 ± 2.24	1.36	3.51
25	SpNO	Y = 10585.85X - 262.99	0.999	0.20	0.50	73.51 ± 1.42	79.47 ± 1.21	80.19 ± 1.05	2.44	4.20
26	Sv	Y = 19665.18X - 1888.36	0.999	0.05	0.20	83.60 ± 0.25	82.63 ± 1.11	82.90 ± 1.14	0.75	2.52
27	SvNO	Y = 9624.64X - 455.32	0.999	0.20	0.50	78.02 ± 0.34	89.94 ± 2.53	90.67 ± 5.21	4.29	3.27
28	7-Im	Y = 107254.00X - 6703.07	0.999	0.01	0.10	70.00 ± 1.52	81.60 ± 1.26	82.67 ± 1.26	1.17	0.14
29	7-ImNO	Y = 30615.70X - 1674.86	0.999	0.05	0.20	70.45 ± 1.29	71.42 ± 0.94	71.52 ± 0.75	4.42	4.50
30	Sk	Y = 42026.84X - 2164.34	0.998	0.02	0.10	102.96 ± 3.07	100.08 ± 2.35	90.05 ± 1.56	1.19	1.43

31	Td	Y = 25791.47X - 974.73	0.998	0.05	0.20	70.62 ± 0.24	90.04 ± 0.04	84.26 ± 0.17	3.33	3.95
32	7-Ly	Y = 63129.24X - 7460.31	0.995	0.01	0.10	70.65 ± 1.53	83.40 ± 1.48	80.49 ± 0.04	0.45	1.04

Table S8. The PA content detected in all 386 herbal medicines, and the corresponding EDI and MOE values

NO.	Herbal samples Latin name	Collection source*	Number of PAs detected and top three	Total PAs content (μg/kg)	Herbals maximum comsumption in the ChP (2020) (g/day)	PAs maximum consumption (μg/day)	EDI (μg/kg bw/day).	MOE values for daily lifetime exposure	EDI (2 weeks every year during a lifetime exposure) = EDI/26	MOE values for 2 weeks every year during a lifetime exposure
HM-1	*Arnebia euchroma* (Royle) Johnst	1, 2, 3	7 (LyNO,ImNO,EmNO)	25567.4	10	255.674	3.652491429	64.88721593	0.14048044	1687.067614
HM-2	*Tussilago farfara* L.	1, 2, 3	10 (Sk,SnNO,Sn)	17600.2	10	176.002	2.514311429	94.26039961	0.096704286	2450.77039
HM-3	*Eupatorium fortunei* Turcz.	1, 3	5 (Im,Ly,LyNO)	16943.7	10	169.437	2.420522857	97.9127296	0.093097033	2545.73097
HM-4	*Eupatorium lindleyanum* DC.	3	5 (LyNO,Im,Ly)	1863.68	60	111.821	1.59744	148.3623798	0.06144	3857.421875
HM-5	*Senecio scandens* Buch.-Ham.	3	12 (Sk,SnN0,SpNO)	229.03	30	6.8708	0.098155714	2414.530847	0.00377522	62777.80203
HM-6	*Laggera pterodonta* (DC.) Benth.	3	9 (Im,ImNO,JbNO)	198.58	15	2.9787	0.042552857	5569.543761	0.001636648	144808.1378
HM-7	*Artemisia scoparia* Waldst. *et* Kit.	1, 2, 3	6 (Im,Ly,LyNO)	233.63	10	3.5045	0.033375714	7100.971622	0.001283681	184625.2622

HM-8	*Cassia angustifolia* Vahl	1, 2, 3	9 (Mc,McNO,Td)	220.69	6	1.3241	0.018916286	12528.88667	0.000727549	325751.0535
HM-9	*Euphorbia hirta* L.	3	6 (Im,ImNO,LyNO)	121.9	9	1.0971	0.015672857	15121.68444	0.000602802	393163.7955
HM-10	*Gynura japonica* (Thunb.) Juel.	1, 2, 3	6 (SnNO,SpNO,Sk)	531.041	6	3.1862	0.045517758	5206.759129	0.001750683	135375.7374
HM-11	*Scutellaria barbata* D.Don	1	5 (LyNO,Ly,ImNO)	61.73	30	1.852	0.026455714	8958.367082	0.001017527	232917.5441
HM-12	*Euphorbia humifusa* Willd.	1, 2, 3	6 (LyNO,Ly,ImNO)	45.03	20	0.9005	0.012865714	18421.05263	0.000494835	478947.3684
HM-13	*Picria fel-terrae* Lour.	3	6 (LyNO,Ly,Im)	58.26	15	0.8739	0.012484286	18983.86543	0.000480165	493580.5012
HM-14	*Cirsium japonicum* Fisch. ex DC.	1, 2, 3	7 (Im,Sk,LyNO)	44.6	15	0.6691	0.009557143	24798.20628	0.000367582	644753.3632
HM-15	*Abrus cantoniensis* Hance	1, 2, 3	6 (LyNO,Ly,ImNO)	22.06	30	0.6619	0.009454286	25067.99637	0.000363626	651767.9057
HM-16	*Equisetum hyemale* L.	1	6 (LyNO,Ly,ImNO)	66.6	9	0.5994	0.008562857	27677.67768	0.000329341	719619.6196
HM-17	*Apocynum venetum* L.	1, 2, 3	5 (LyNO,Ly,ImNO)	49.25	12	0.591	0.008442857	28071.06599	0.000324725	729847.7157
HM-18	*Siphonostegia chinensis* Benth.	1, 2, 3	13 (Im,ImNO,LyNO)	51.88	9	0.467	0.006670286	35530.7119	0.000256549	923798.5094

HM-19	*Achillea alpina* L.	3	4 (Sk,Im,Ly)	51.9	45	2.3356	0.033364286	7103.403982	0.001283242	184688.5035
HM-20	*Phryma leptostachya* L. *subsp. asiatica* (Hara) Kitamura	1, 2, 3	6 (LyNO,Ly,ImNO)	45.7409	15	0.6861	0.009801621	24179.6729	0.000376985	628671.4953
HM-21	*Hedyotis diffusa*	1		7.2403	30	0.2172	0.003102986	76378.05063	0.000119346	1985829.316
HM-22	*Angelica sinensis*	1, 2, 3		1.0625	10	0.0106	0.000151786	1561411.765	5.83791E-06	40596705.88
HM-23	*Thlaspi arvense* Linn.	1, 2, 3		14.139	30	0.4242	0.006059571	39111.67692	0.00023306	1016903.6
HM-24	*Boehmeria nivea* (L.) Gaudich.	3		0.4974	10	0.0050	7.10571E-05	3335343.788	2.73297E-06	86718938.48
HM-25	*Triticum aestivum* L.	3		0.8149	10	0.0081	0.000116414	2035832.617	4.47747E-06	52931648.05
HM-26	*Zea mays* L.	1		0.9141	10	0.0091	0.000130586	1814899.902	5.02253E-06	47187397.44
HM-27	*Benincasa hispida* (Thunb.) Cogn.	2		0.0962	10	0.0010	1.37429E-05	17245322.25	5.28571E-07	448378378.4
HM-28	*Pteris multifida* Poir.	2		0.9641	10	0.0096	0.000137729	1720775.853	5.29725E-06	44740172.18

HM-29	*Gynostemma pentaphyllum* (Thunb.) Makino	1, 2	1.3948	10	0.0139	0.000199257	1189417.838	7.66374E-06	30924863.78
HM-30	*Ramulus Euonymi*	1	0.2362	10	0.0024	3.37429E-05	7023708.721	1.2978E-06	182616426.8
HM-31	*Paederia scandens* (Lour.) Merr.	1, 3	0.232	10	0.0023	3.31429E-05	7150862.069	1.27473E-06	185922413.8
HM-32	*Ilex pubescens* Hook. *et* Arn.	1, 2	0.5073	10	0.0051	7.24714E-05	3270254.287	2.78736E-06	85026611.47
HM-33	*Pogostemon cablin* (Blanco) Benth.	2	40.75	10	0.4075	0.005821429	40711.65644	0.000223901	1058503.067
HM-34	*Eclipta prostrata* L.	1, 2, 3	29.44	12	0.3532	0.005046857	46959.91848	0.00019411	1220957.88
HM-35	*Taraxacum mongolicum* Hand.-Mazz.	1, 2, 3	22.71	15	0.3407	0.004866429	48701.01277	0.00018717	1266226.332
HM-36	*Erigeron breviscapus* (Vant.) Hand.-Mazz.	1, 3	20.86	15	0.313	0.00447	53020.13423	0.000171923	1378523.49
HM-37	*Arctium lappa* L.	1, 3	25.42	12	0.3051	0.004357714	54386.30999	0.000167604	1414044.06

HM-38	*Solidago decurrens* Lour.	3	19.39	15	0.2909	0.004155	57039.71119	0.000159808	1483032.491
HM-39	*Desmodium styracifolium* (Osb.) Merr.	1	9.52	30	0.2856	0.00408	58088.23529	0.000156923	1510294.118
HM-40	*Imperata cylindrica* Beauv. *var.* major (Nees) C. E. Hubb.	1, 2, 3	8.44	30	0.2531	0.003617143	65521.32701	0.000139121	1703554.502
HM-41	*Clematis chinensis* Osbeck.	1	25.17	10	0.2517	0.003595714	65911.79976	0.000138297	1713706.794
HM-42	*Dalbergia odorifera* T. Chen	1, 2, 3	16.24	15	0.2437	0.00348	68103.44828	0.000133846	1770689.655
HM-43	*Inula linariifolia* Turcz.	1, 3	23.24	10	0.2324	0.00332	71385.54217	0.000127692	1856024.096
HM-44	*Vaccaria segetalis* (Neck.) Garcke	1	22.81	10	0.2281	0.003258571	72731.25822	0.00012533	1891012.714
HM-45	*Albizia julibrissin* Durazz.	1, 2, 3	15.62	12	0.1874	0.002677714	88508.32266	0.000102989	2301216.389
HM-46	*Atractylodes lancea* (Thunb.) DC.	1, 3	20.71	9	0.1864	0.002662714	89006.92097	0.000102412	2314179.945

HM-47	*Inula helenium* L.	3	18.12	9	0.1631	0.002329714	101729.2127	8.96044E-05	2644959.529
HM-48	*Pinus tabulieformis* Carr.	1	10.46	15	0.1568	0.002241429	105736.1377	8.62088E-05	2749139.579
HM-49	*Mosla chinensis* Maxim.	1	14.75	10	0.1475	0.002107143	112474.5763	8.1044E-05	2924338.983
HM-50	*Cirsium setosum* (Willd.) MB.	1, 2, 3	12.02	12	0.1443	0.002060571	115016.6389	7.92527E-05	2990432.612
HM-51	*Aster tataricus* L. f.	1, 3	13.89	10	0.1389	0.001984286	119438.4449	7.63187E-05	3105399.568
HM-52	*Artemisia annua* L.	1, 2, 3	11.04	12	0.1324	0.001892571	125226.4493	7.27912E-05	3255887.681
HM-53	*Siegesbeckia orientalis* L.	1, 3	10.95	12	0.1313	0.001877143	126255.7078	7.21978E-05	3282648.402
HM-54	*Aucklandia lappa* Decne.	1, 2, 3	18.49	6	0.111	0.001584857	149540.292	6.0956E-05	3888047.593
HM-55	*Smilax glabra* Roxb.	1	1.76	60	0.1057	0.001508571	157102.2727	5.8022E-05	4084659.091
HM-56	*Astragalus propinquus* Schischkin	1,2	3.42	30	0.1025	0.001464046	161880.1166	5.63095E-05	4208883.033

HM-57	*Caesalpinia sappan L.*	1, 2, 3		11.07	9	0.0996	0.001423286	166516.1096	5.47418E-05	4329418.85
HM-58	*Rehmannia glutinosa* Libosch.	1, 2, 3		6.41	15	0.0962	0.001373571	172542.9017	5.28297E-05	4486115.445
HM-59	*Coix lacryma-jobi* L. *var.* ma-yuen (Roman.) Stapf	1, 2, 3		3.2	30	0.0959	0.001371429	172812.5	5.27473E-05	4493125
HM-60	*Carthamus tinctorius* L.	1, 2, 3		9.45	10	0.0945	0.00135	175555.5556	5.19231E-05	4564444.444
HM-61	*Ampelopsis japonica* (Thunb.) Makino	1		8.16	10	0.0816	0.001165714	203308.8235	4.48352E-05	5286029.412
HM-62	*Glycine max* (L.) Merr.	1, 3		5.42	15	0.0813	0.001161429	204059.0406	4.46703E-05	5305535.055
HM-63	*Echinops latifolius* Tausch.	3		8.1	10	0.081	0.001157143	204814.8148	4.45055E-05	5325185.185
HM-64	*Inula japonica* Thunb.	1, 2, 3		8.73	9	0.0786	0.001122429	211149.2936	4.31703E-05	5489881.634
HM-65	*Trachelospermum jasminoides* (Lindl.) Lem.	1, 2, 3		5.77	12	0.0693	0.000989143	239601.3865	3.8044E-05	6229636.049

HM-66	*Artemisia argyi* Levl. *et* Vant.	1, 3	7.53	9	0.0678	0.000968143	244798.5834	3.72363E-05	6364763.17
HM-67	*Pulsatilla chinensis* (Bge.) Regel	1	4.36	15	0.0653	0.000934286	253669.7248	3.59341E-05	6595412.844
HM-68	*Xanthium sibiricum* Patr.	1, 3	6.53	10	0.0653	0.000932857	254058.193	3.58791E-05	6605513.017
HM-69	*Hordeum vulgare* L.	1, 2, 3	4.28	15	0.0643	0.000917143	258411.215	3.52747E-05	6718691.589
HM-70	*Sauropus spatulifolius* Beille	3	4.16	15	0.0623	0.000891429	265865.3846	3.42857E-05	6912500
HM-71	*Phyllanthus emblica* L.	3	6.45	9	0.058	0.000829286	285788.1137	3.18956E-05	7430490.956
HM-72	*Bupleurum chinense* DC.	1	5.7	10	0.057	0.000814286	291052.6316	3.13187E-05	7567368.421
HM-73	*Bletilla striata* (Thunb.) Reichb. f.	1, 2, 3	3.69	15	0.0554	0.000790714	299728.9973	3.04121E-05	7792953.93
HM-74	*Fagopyrum dibotrys* (D. Don) Ham	1	1.13	45	0.051	0.000726429	326253.6873	2.79396E-05	8482595.87

HM-75	*Sophora japonica* L.	1, 3	4.88	10	0.0488	0.000697143	339959.0164	2.68132E-05	8838934.426
HM-76	*Corydalis bungeana* Turcz.	1	3.13	15	0.0469	0.000670714	353354.6326	2.57967E-05	9187220.447
HM-77	*Adenophora stricta* Miq.	1	3.1	15	0.0465	0.000664286	356774.1935	2.55495E-05	9276129.032
HM-78	*Carpesium abrotanoides* L.	1, 3	5.09	9	0.0458	0.000654429	362148.0026	2.51703E-05	9415848.068
HM-79	*Ghrysanthemum indicum* L.	1, 2, 3	2.95	15	0.0442	0.000632143	374915.2542	2.43132E-05	9747796.61
HM-80	*Albizia julibrissin* Durazz.	1, 3	4.24	10	0.0424	0.000605714	391273.5849	2.32967E-05	10173113.21
HM-81	*Vigna umbellata* Ohwi *et* Ohashi	1, 2, 3	1.4	30	0.0421	0.0006	395000	2.30769E-05	10270000
HM-82	*Rosa rugosa* Thunb.	1	6.98	6	0.0419	0.000598286	396131.8052	2.3011E-05	10299426.93
HM-83	*Epimedium koreanum* Nakai	1	3.83	10	0.0383	0.000547143	433159.2689	2.1044E-05	11262140.99
HM-84	*Lycopodium japonicum* Thunb.	1	3.07	12	0.0369	0.000526286	450325.7329	2.02418E-05	11708469.06

HM-85	*Canavalia gladiata* (Jacq.) DC.	1, 3		3.75	9	0.0337	0.000482143	491555.5556	1.8544E-05	12780444.44
HM-86	*Ophiopogon japonicus* (L. f) Ker-GawL	1		2.78	12	0.0334	0.000476571	497302.1583	1.83297E-05	12929856.12
HM-87	*Allium macrostemon* Bge.	1		3.26	10	0.0326	0.000465714	508895.7055	1.79121E-05	13231288.34
HM-88	*Morus alba* L.	1		2.17	15	0.0325	0.000465	509677.4194	1.78846E-05	13251612.9
HM-89	*Andrographis paniculata* (Burm.f.) Nees	1		3.35	9	0.0302	0.000430714	550248.7562	1.65659E-05	14306467.66
HM-90	*Acanthoppanax gracilistylus* W. W. Smith.	1		2.9	10	0.029	0.000414286	572068.9655	1.59341E-05	14873793.1
HM-91	*Physalis alkekengi* L. *var.* franchetii (Mast.) Makino	1		3.19	9	0.0287	0.000410143	577847.4399	1.57747E-05	15024033.44

HM-92	*Rhaponticum uniflorum* (L.) DC.	1, 3	3.18	9	0.0286	0.000408857	579664.5702	1.57253E-05	15071278.83
HM-93	*Erodium stephanianum* Willd.	1	1.88	15	0.0283	0.000402857	588297.8723	1.54945E-05	15295744.68
HM-94	*Polygonum aviculare* L.	1	1.86	15	0.0279	0.000398571	594623.6559	1.53297E-05	15460215.05
HM-95	*Luffa cylindrica* (L.) Roem.	1	2.31	12	0.0277	0.000396	598484.8485	1.52308E-05	15560606.06
HM-96	*Sophora tonkinensis* Gagnep.	1, 2, 3	4.54	6	0.0272	0.000389143	609030.837	1.4967E-05	15834801.76
HM-97	*Sarcandra glabra* (Thunb.) Nakai	1	0.89	30	0.0266	0.000381429	621348.3146	1.46703E-05	16155056.18
HM-98	*Vitex trifolia* L. *var.* simplicifolia Cham.	1	2.53	10	0.0253	0.000361429	655731.2253	1.39011E-05	17049011.86
HM-99	*Glycyrrhiza uralensis* Fisch.	1, 2, 3	2.47	10	0.0247	0.000352857	671659.919	1.35714E-05	17463157.89

HM-100	*Paris polyphylla* Smith *var.* chinensis (Franch.) Hara	1		2.54	9	0.0228	0.000326571	725721.7848	1.25604E-05	18868766.4
HM-101	*Gastrodia elata* Bl.	1, 2, 3		2.19	10	0.0219	0.000312857	757534.2466	1.2033E-05	19695890.41
HM-102	*Homalomena occulta* (Lour.) Schott	1		2.16	10	0.0216	0.000308571	768055.5556	1.18681E-05	19969444.44
HM-103	*Plantago asiatica* L. *&* Plantago depressa Willd.	1		0.68	30	0.0203	0.000291429	813235.2941	1.12088E-05	21144117.65
HM-104	*Paeonia lactiflora* PalL	1, 2		1.68	12	0.0201	0.000288	822916.6667	1.10769E-05	21395833.33
HM-105	*Glycine max* (L.) Merr.	1, 2, 3		0.67	30	0.0201	0.000287143	825373.1343	1.1044E-05	21459701.49
HM-106	*Paeonia suffruticosa* Andr.	1, 2		1.64	12	0.0196	0.000281143	842987.8049	1.08132E-05	21917682.93
HM-107	*Dolichos lablab* L.	1, 2, 3		1.26	15	0.019	0.00027	877777.7778	1.03846E-05	22822222.22

HM-108	*Centipeda minima* (L.) A.Br. *et* Aschers.	1, 2, 3	2.05	9	0.0185	0.000263571	899186.9919	1.01374E-05	23378861.79
HM-109	*Gentiana macrophylla* Pall.	1	1.67	10	0.0167	0.000238571	993413.1737	9.17582E-06	25828742.51
HM-110	*Verbena Officinalis* L.	1	1.67	10	0.0167	0.000238571	993413.1737	9.17582E-06	25828742.51
HM-111	*Scrophularia ningpoensis* Hemsl.	1, 2, 3	1.1	15	0.0166	0.000235714	1005454.545	9.06593E-06	26141818.18
HM-112	*Picrorhiza scrophulariiflora* Pennell	1, 2, 3	1.65	10	0.0165	0.000235714	1005454.545	9.06593E-06	26141818.18
HM-113	*Akebia quinata* (Thunb.) Decne.	1	1.8	9	0.0162	0.000231429	1024074.074	8.9011E-06	26625925.93
HM-114	*Cuscuta australis* R. Br.	1	1.35	12	0.0161	0.000231429	1024074.074	8.9011E-06	26625925.93
HM-115	*Gardenia jasminoides* Ellis	1	1.61	10	0.0161	0.00023	1030434.783	8.84615E-06	26791304.35
HM-116	*Acacia catechu* (L. f.) Willd.	1, 3	5.22	3	0.0157	0.000223714	1059386.973	8.6044E-06	27544061.3

HM-117	*Notopterygium inchum* Ting *ex* H, T- Chang	1	1.56	10	0.0156	0.000222857	1063461.538	8.57143E-06	27650000
HM-118	*Kaempferia galanga* L.	1	1.66	9	0.015	0.000213429	1110441.767	8.20879E-06	28871485.94
HM-119	*Sophora flavescens* Ait.	1, 2, 3	1.65	9	0.0148	0.000212143	1117171.717	8.15934E-06	29046464.65
HM-120	*Setaria italica* (L.) Beauv.	1, 2, 3	0.98	15	0.0147	0.00021	1128571.429	8.07692E-06	29342857.14
HM-121	*Astragalus membranaceus* (Fisch.) Bge.	1, 2, 3	0.49	30	0.0146	0.00021	1128571.429	8.07692E-06	29342857.14
HM-122	*Lophatherum gracile* Brongn.	1, 2, 3	1.41	10	0.0141	0.000201429	1176595.745	7.74725E-06	30591489.36
HM-123	*Dendrobium nobile* Lindl	1, 2, 3	1.14	12	0.0136	0.000195429	1212719.298	7.51648E-06	31530701.75
HM-124	*Eriocaulon buergerianum* Koern.	1	1.36	10	0.0136	0.000194286	1219852.941	7.47253E-06	31716176.47
HM-125	*Phragmites communis* Trin.	1, 2, 3	0.45	30	0.0134	0.000192857	1228888.889	7.41758E-06	31951111.11

HM-126	*Spatholobus suberectus* Dunn	1, 2, 3	0.89	15	0.0133	0.000190714	1242696.629	7.33516E-06	32310112.36
HM-127	*Santalum album* L.	1, 3	2.53	5	0.0126	0.000180714	1311462.451	6.95055E-06	34098023.72
HM-128	*Cralaegus pinnatifida.* Bge. *var. major* N.E.Br.	1	1.03	12	0.0123	0.000176571	1342233.01	6.79121E-06	34898058.25
HM-129	*Lysimachia christinae* Hance	2	0.2	60	0.0119	0.000171429	1382500	6.59341E-06	35945000
HM-130	*Ranunculus ternatus* Thunb.	1	0.39	30	0.0118	0.000167143	1417948.718	6.42857E-06	36866666.67
HM-131	*Cichorium glandulosum* Boiss.*et* Huet	3	0.65	18	0.0116	0.000167143	1417948.718	6.42857E-06	36866666.67
HM-132	*Polygonum multiflorum* Thumb.	1	0.75	15	0.0113	0.000160714	1474666.667	6.18132E-06	38341333.33
HM-133	*Stachyurus himalai-cus* Hook.f.et Thoms.	1	1.85	6	0.0111	0.000158571	1494594.595	6.0989E-06	38859459.46
HM-134	*Dimocarpus longan* Lour.	1	0.73	15	0.0109	0.000156429	1515068.493	6.01648E-06	39391780.82

HM-135	*Pueraria lobata* (Willd.) Ohwi	1, 2, 3		0.69	15	0.0104	0.000147857	1602898.551	5.68681E-06	41675362.32
HM-136	*Curcuma Longa* L.	1		1.03	10	0.0103	0.000147143	1610679.612	5.65934E-06	41877669.9
HM-137	*Selaginella tamariscina* (Beauv.) Spring	1		1.01	10	0.0101	0.000144286	1642574.257	5.54945E-06	42706930.69
HM-138	*Ilex cornuta* Lindl. *ex* Paxt.	1		0.63	15	0.0094	0.000135	1755555.556	5.19231E-06	45644444.44
HM-139	*Paeonia lactiflora* Pall.	1		0.6	15	0.009	0.000128571	1843333.333	4.94505E-06	47926666.67
HM-140	*Lobelia chinensis* Lour.	1		0.59	15	0.0089	0.000126429	1874576.271	4.86264E-06	48738983.05
HM-141	*Fraxinus rhynchophylla* Hance	1		0.72	12	0.0087	0.000123429	1920138.889	4.74725E-06	49923611.11
HM-142	*Euphorbia pekinensis* Rupr.	3		2.88	3	0.0086	0.000123429	1920138.889	4.74725E-06	49923611.11
HM-143	*Atractylodes macrocephala* Koidz.	1, 2, 3		0.72	12	0.0086	0.000123429	1920138.889	4.74725E-06	49923611.11

HM-144	*Cassia obtusifolia* L.	1, 2, 3	0.56	15	0.0085	0.00012	1975000	4.61538E-06	51350000
HM-145	*Piper kadsura* (CHOisy) Ohwi	1	0.7	12	0.0084	0.00012	1975000	4.61538E-06	51350000
HM-146	*Curcuma kwangsiensis* S. G. Lee *et* C. F. Liang	1	0.84	10	0.0084	0.00012	1975000	4.61538E-06	51350000
HM-147	*Lycium barbarum* L.	1	0.7	12	0.0084	0.00012	1975000	4.61538E-06	51350000
HM-148	*Sesamum indicum* L.	2	0.56	15	0.0083	0.00012	1975000	4.61538E-06	51350000
HM-149	*Citrus medica* L.	1	0.83	10	0.0083	0.000118571	1998795.181	4.56044E-06	51968674.7
HM-150	*Glycine max* (L.) Merr.	1, 2, 3	0.68	12	0.0082	0.000116571	2033088.235	4.48352E-06	52860294.12
HM-151	*Salvia miltiorrhiza* Bge.	1	0.54	15	0.0082	0.000115714	2048148.148	4.45055E-06	53251851.85
HM-152	*Leonurus japonicus* Houtt.	1	0.27	30	0.0081	0.000115714	2048148.148	4.45055E-06	53251851.85
HM-153	*Polygonatum ordoratum* (Mill.) Druce	1	0.67	12	0.0081	0.000114857	2063432.836	4.41758E-06	53649253.73

HM-154	*Aquilaria sinensis* (Lour.) Gilg	1		1.6	5	0.008	0.000114286	2073750	4.3956E-06	53917500
HM-155	*Cornus officinalis* Sieb. *et* Zucc.	1		0.66	12	0.0079	0.000113143	2094696.97	4.35165E-06	54462121.21
HM-156	*Angelica dahurica* (Fisch. ex Hoffm.) Benth. *et* Hook. f.	1		0.79	10	0.0079	0.000112857	2100000	4.34066E-06	54600000
HM-157	*Lonicera japonica* Thunb.	1		0.53	15	0.0079	0.000113571	2086792.453	4.36813E-06	54256603.77
HM-158	*Nigella glandulifera* Freyn *et* Sint.	3		1.31	6	0.0079	0.000112286	2110687.023	4.31868E-06	54877862.6
HM-159	*Codonopsis pilosula* (Franch.) Nannf.	1		0.26	30	0.0078	0.000111429	2126923.077	4.28571E-06	55300000
HM-160	*Panax quinquefolium* L.	1		1.29	6	0.0077	0.000110571	2143410.853	4.25275E-06	55728682.17
HM-161	*Houttuynia cordata* Thunb.	1		0.31	25	0.0076	0.000110714	2140645.161	4.25824E-06	55656774.19

HM-162	*Gleditsia sinensis* Lam.	1, 2, 3	0.76	10	0.0076	0.000108571	2182894.737	4.17582E-06	56755263.16
HM-163	*Prunus mume* (Sieb.) Sieb. *et* Zucc.	1	1.5	5	0.0075	0.000107143	2212000	4.12088E-06	57512000
HM-164	*Citrus medica* L. *var*. sarcodactylis Swingle	1	0.74	10	0.0074	0.000105714	2241891.892	4.06593E-06	58289189.19
HM-165	*Asparagus cochinchinensis* (Lour.) Merr.	1	0.6	12	0.0072	0.000102857	2304166.667	3.95604E-06	59908333.33
HM-166	*Zingiber officinale* Rosc.	1	0.8	9	0.0072	0.000102857	2304166.667	3.95604E-06	59908333.33
HM-167	*Sanguisorba officinalis* L.	1	0.48	15	0.0072	0.000102857	2304166.667	3.95604E-06	59908333.33
HM-168	*Isatis indigotica* Fort.	1	0.48	15	0.0071	0.000102857	2304166.667	3.95604E-06	59908333.33
HM-169	*Mentha haplocalyx* Briq.	1	1.16	6	0.007	9.94286E-05	2383620.69	3.82418E-06	61974137.93
HM-170	*Gremastra appendiculata* (D.Don) Makino	1, 3	0.72	9	0.0064	9.25714E-05	2560185.185	3.56044E-06	66564814.81

HM-171	*Gentiana manshuric* Kitag.	1	1.06	6	0.0064	9.08571E-05	2608490.566	3.49451E-06	67820754.72
HM-172	*Platycodon grandiflorum* (Jacq.) A. DC.	1	0.62	10	0.0062	8.85714E-05	2675806.452	3.40659E-06	69570967.74
HM-173	*Vladimiria souliei* (Franch.) Ling	3	0.66	9	0.006	8.48571E-05	2792929.293	3.26374E-06	72616161.62
HM-174	*Cynanchum atratum* Bge.	1	0.6	10	0.006	8.57143E-05	2765000	3.2967E-06	71890000
HM-175	*Cimicifuga heracleifolia* Kom.	1, 2	0.59	10	0.0059	8.42857E-05	2811864.407	3.24176E-06	73108474.58
HM-176	*Cynanchum stauntonii* (Decne.) Schltr. *ex* Levi.	1	0.59	10	0.0059	8.42857E-05	2811864.407	3.24176E-06	73108474.58
HM-177	*Bambusa tuldoides* Munro	1, 2, 3	0.57	10	0.0057	8.14286E-05	2910526.316	3.13187E-06	75673684.21
HM-178	*Dipsacus asper* Wall. *ex* Henry	1	0.37	15	0.0055	7.92857E-05	2989189.189	3.04945E-06	77718918.92
HM-179	*Morinda officinalis* How	1	0.54	10	0.0054	7.71429E-05	3072222.222	2.96703E-06	79877777.78

HM-180	*Lilium lancifolium* Thunb.	1	0.45	12	0.0054	7.71429E-05	3072222.222	2.96703E-06	79877777.78
HM-181	*Magnolia biondii* Pamp.	1	0.53	10	0.0053	7.57143E-05	3130188.679	2.91209E-06	81384905.66
HM-182	*Cyathula officinalis* Kuan	1	0.53	10	0.0053	7.57143E-05	3130188.679	2.91209E-06	81384905.66
HM-183	*Illicium verum* Hook f.	1, 3	0.85	6	0.0051	7.28571E-05	3252941.176	2.8022E-06	84576470.59
HM-184	*Oryza sativa* L.	1, 2, 3	0.33	15	0.005	7.07143E-05	3351515.152	2.71978E-06	87139393.94
HM-185	*Rubus chingii* Hu	1	0.41	12	0.0049	7.02857E-05	3371951.22	2.7033E-06	87670731.71
HM-186	*Prunus mume* (Sieb.) Sieb. *et* Zucc.	1	0.41	12	0.0049	7.02857E-05	3371951.22	2.7033E-06	87670731.71
HM-187	*Alpinia katsumadai* Hayata	1	0.81	6	0.0049	6.94286E-05	3413580.247	2.67033E-06	88753086.42
HM-188	*Liquidambar formosana* Hance	1	0.48	10	0.0048	6.85714E-05	3456250	2.63736E-06	89862500
HM-189	*Alpinia officinarum* Hance	1	0.78	6	0.0047	6.68571E-05	3544871.795	2.57143E-06	92166666.67
HM-190	*Rubia cordifolia* L.	1	0.46	10	0.0046	6.57143E-05	3606521.739	2.52747E-06	93769565.22

HM-191	*Zingiber officinale* Rosc.	1	0.46	10	0.0046	6.57143E-05	3606521.739	2.52747E-06	93769565.22
HM-192	*Cinnamomum cassia* Presl	1	0.91	5	0.0045	0.000065	3646153.846	0.0000025	94800000
HM-193	*Achyranthes bidentata* Bl.	1	0.36	12	0.0044	6.17143E-05	3840277.778	2.37363E-06	99847222.22
HM-194	*Cynanchum paniculatum* (Bge.) Kitag.	1	0.36	12	0.0044	6.17143E-05	3840277.778	2.37363E-06	99847222.22
HM-195	*Raphanus sativus* L.	1	0.36	12	0.0043	6.17143E-05	3840277.778	2.37363E-06	99847222.22
HM-196	*Cyperus rotundus* L.	1	0.42	10	0.0042	0.00006	3950000	2.30769E-06	102700000
HM-197	*Cnidium monnieri* (L.) Cuss.	1	0.41	10	0.0041	5.85714E-05	4046341.463	2.25275E-06	105204878
HM-198	*Asarum heterotropoides* Fr. Schmidt *var.* mandshuricum (Maxim.) Kitag.	1	1.35	3	0.004	5.78571E-05	4096296.296	2.22527E-06	106503703.7

HM-199	*Panax notoginseng* (Burk.) F. H. Chen	1	0.44	9	0.004	5.65714E-05	4189393.939	2.17582E-06	108924242.4
HM-200	*Taxillus chinensis* (DC.) Danser	1	0.26	15	0.0039	5.57143E-05	4253846.154	2.14286E-06	110600000
HM-201	*Potentilla chinensis* Ser.	1	0.26	15	0.0039	5.57143E-05	4253846.154	2.14286E-06	110600000
HM-202	*Ziziphus jujuba* Mill.	1	0.26	15	0.0039	5.57143E-05	4253846.154	2.14286E-06	110600000
HM-203	*Broussonetia papyrifera* (L.) Vent.	1	0.31	12	0.0037	5.31429E-05	4459677.419	2.04396E-06	115951612.9
HM-204	*Tribulus terrestris* L.	1	0.37	10	0.0037	5.28571E-05	4483783.784	2.03297E-06	116578378.4
HM-205	*Citrus aurantium* L.	1	0.37	10	0.0037	5.28571E-05	4483783.784	2.03297E-06	116578378.4
HM-206	*Curculigo orchioides* Gaertn.	1	0.37	10	0.0037	5.28571E-05	4483783.784	2.03297E-06	116578378.4
HM-207	*Panax ginseng* C. A. Mey.	1	0.4	9	0.0036	5.14286E-05	4608333.333	1.97802E-06	119816666.7

HM-208	*Lygodium japonicum* (Thunb.) Sw.	1	0.24	15	0.0036	5.14286E-05	4608333.333	1.97802E-06	119816666.7
HM-209	*Sargentodoxa cuneata* (Oliv.) Rehd. *et* Wils.	1	0.24	15	0.0036	5.14286E-05	4608333.333	1.97802E-06	119816666.7
HM-210	*Morus alba* L.	1	0.24	15	0.0036	5.14286E-05	4608333.333	1.97802E-06	119816666.7
HM-211	*Eriobotrya japonica* (Thunb.) Lindl.	1	0.35	10	0.0035	0.00005	4740000	1.92308E-06	123240000
HM-212	*Dendrobium officinale* Kimura *et* Migo	1, 3	0.28	12	0.0034	0.000048	4937500	1.84615E-06	128375000
HM-213	*Trichosanthes kirilowii* Maxim.	1	0.22	15	0.0034	4.71429E-05	5027272.727	1.81319E-06	130709090.9
HM-214	*Leonurus japonicus* Houtt.	1	0.33	10	0.0033	4.71429E-05	5027272.727	1.81319E-06	130709090.9
HM-215	*Magnolia officinalis* Rehd.*et* Wils.	1	0.36	9	0.0032	4.62857E-05	5120370.37	1.78022E-06	133129629.6

HM-216	*Terminalia chebula* Retz.	1	0.32	10	0.0032	4.57143E-05	5184375	1.75824E-06	134793750
HM-217	*Celosia argentea* L.	1	0.21	15	0.0032	0.000045	5266666.667	1.73077E-06	136933333.3
HM-218	*Forsythia suspensa* (Thunb.) Vahl	1	0.21	15	0.0031	0.000045	5266666.667	1.73077E-06	136933333.3
HM-219	*Saussurea involucrata* (Kar.*et* Kir.) Sch.-Bip.	1, 3	0.52	6	0.0031	4.45714E-05	5317307.692	1.71429E-06	138250000
HM-220	*Unacaria rhynchophylla* (Miq.) Miq. *ex* Havil.	1	0.25	12	0.003	4.28571E-05	5530000	1.64835E-06	143780000
HM-221	*Cibotium barometz* (L.) J. Sm.	1	0.23	12	0.0028	3.94286E-05	6010869.565	1.51648E-06	156282608.7
HM-222	*Myristica fragrans* Houtt.	1	0.28	10	0.0028	0.00004	5925000	1.53846E-06	154050000
HM-223	*Laminaria japonica* Aresch.	1	0.23	12	0.0028	3.94286E-05	6010869.565	1.51648E-06	156282608.7
HM-224	*Dioscorea nipponica* Makino	1	0.18	15	0.0028	3.85714E-05	6144444.444	1.48352E-06	159755555.6

HM-225	*Nardostachys jatamansi* DC.	1	0.44	6	0.0027	3.77143E-05	6284090.909	1.45055E-06	163386363.6
HM-226	*Psoralea corylifolia* L.	1, 2, 3	0.26	10	0.0026	3.71429E-05	6380769.231	1.42857E-06	165900000
HM-227	*Sinapis alba* L.	1	0.29	9	0.0026	3.72857E-05	6356321.839	1.43407E-06	165264367.8
HM-228	*Eucommia ulmoides* Oliv.	1	0.25	10	0.0025	3.57143E-05	6636000	1.37363E-06	172536000
HM-229	*Citrus reticulata* Blanco	1	0.25	10	0.0025	3.57143E-05	6636000	1.37363E-06	172536000
HM-230	*Plantago asiatica* L. & Plantago depressa Willd.	1	0.16	15	0.0024	3.42857E-05	6912500	1.31868E-06	179725000
HM-231	*Perilla frutescem* (L.) Britt.	1	0.24	10	0.0024	3.42857E-05	6912500	1.31868E-06	179725000
HM-232	*Dianthus superbus* L. & Dianthus chinensis L.	1	0.16	15	0.0024	3.42857E-05	6912500	1.31868E-06	179725000
HM-233	*Curcuma phaeocaulis* Val.	2	0.26	9	0.0023	3.34286E-05	7089743.59	1.28571E-06	184333333.3

HM-234	*Ephedra sinica* Stapf .	1	0.23	10	0.0023	3.28571E-05	7213043.478	1.26374E-06	187539130.4
HM-235	*Ricinus communis* L.	1, 3	0.45	5	0.0023	3.21429E-05	7373333.333	1.23626E-06	191706666.7
HM-236	*Clematis armandii* Franch.	1	0.35	6	0.0021	0.00003	7900000	1.15385E-06	205400000
HM-237	*Astragalus complanatus* R. Br.	1, 2, 3	0.13	15	0.0019	2.78571E-05	8507692.308	1.07143E-06	221200000
HM-238	*Siraitia grosvenorii* (Swingle) C. Jeffrey *ex* A. M. Lu *et* Z. Y. Zhang	1	0.13	15	0.0019	2.78571E-05	8507692.308	1.07143E-06	221200000
HM-239	*Canarium album* Raeusch.	1	0.19	10	0.0019	2.71429E-05	8731578.947	1.04396E-06	227021052.6
HM-240	*Stephania tetrandra* S. Moore	1	0.19	10	0.0019	2.71429E-05	8731578.947	1.04396E-06	227021052.6
HM-241	*Cannabis sativa* L.	1	0.12	15	0.0018	2.57143E-05	9216666.667	9.89011E-07	239633333.3
HM-242	*Tinospora sagittata* (Oliv.) Gagnep.	1	0.19	9	0.0018	2.44286E-05	9701754.386	9.3956E-07	252245614
HM-243	*Pyrola calliantha* H. Andres	1	0.11	15	0.0016	2.35714E-05	10054545.45	9.06593E-07	261418181.8
HM-244	*Pueraria thomsonii* Benth.	3	0.11	15	0.0016	2.35714E-05	10054545.45	9.06593E-07	261418181.8

HM-245	*Crocus sativus* L.	1	0.54	3	0.0016	2.31429E-05	10240740.74	8.9011E-07	266259259.3
HM-246	*Impatiens balsamina* L.	1	0.32	5	0.0016	2.28571E-05	10368750	8.79121E-07	269587500
HM-247	*Schizonepeta tenuisfolia* Briq.	1	0.15	10	0.0015	2.14286E-05	11060000	8.24176E-07	287560000
HM-248	*Trichosanthes kirilowii* Maxim.	1	0.1	15	0.0014	2.14286E-05	11060000	8.24176E-07	287560000
HM-249	*Schizonepeta tenuifolia* Briq.	1	0.14	10	0.0014	0.00002	11850000	7.69231E-07	308100000
HM-250	*Morus alba* L.	1	0.14	10	0.0014	0.00002	11850000	7.69231E-07	308100000
HM-251	*Gleditsia sinensis* Lam.	1, 3	0.88	1.5	0.0013	1.88571E-05	12568181.82	7.25275E-07	326772727.3
HM-252	*Rosa laevigata* Michx.	1	0.11	12	0.0013	1.88571E-05	12568181.82	7.25275E-07	326772727.3
HM-253	*Sophora japonica* L.	1, 3	0.14	9	0.0013	0.000018	13166666.67	6.92308E-07	342333333.3
HM-254	*Typhonium giganteum* Engl.	1	0.2	6	0.0012	1.71429E-05	13825000	6.59341E-07	359450000
HM-255	*Cinnamomum cassia* Presl	1	0.12	10	0.0012	1.71429E-05	13825000	6.59341E-07	359450000

HM-256	*Pyrrosia sheareri* (Bak.)	1	0.09	12	0.0011	1.54286E-05	15361111.11	5.93407E-07	399388888.9
HM-257	*Cynomorium songaricum* Rupr.	1	0.11	10	0.0011	1.57143E-05	15081818.18	6.04396E-07	392127272.7
HM-258	*Buddieja officinalis* Maxim.	1	0.12	9	0.0011	1.54286E-05	15361111.11	5.93407E-07	399388888.9
HM-259	*Amomurn kravanh* Pierre *ex* Gagnep.	1	0.17	6	0.001	1.45714E-05	16264705.88	5.6044E-07	422882352.9
HM-260	*Citrus grandis* 'Tomentosa'	1	0.17	6	0.001	1.45714E-05	16264705.88	5.6044E-07	422882352.9
HM-261	*Rhus chinensis* Mill.	1	0.16	6	0.001	1.37143E-05	17281250	5.27473E-07	449312500
HM-262	*Abutilon theophrasti* Medic.	2	0.1	9	0.0009	1.28571E-05	18433333.33	4.94505E-07	479266666.7
HM-263	*Trichosanthes kirilowii* Maxim.	1	0.09	10	0.0009	1.28571E-05	18433333.33	4.94505E-07	479266666.7
HM-264	*Foeniculum vulgare* Mill.	1	0.14	6	0.0008	0.000012	19750000	4.61538E-07	513500000
HM-265	*Gleditsia sinensis* Lam.	1, 2, 3	0.49	1.5	0.0007	0.0000105	22571428.57	4.03846E-07	586857142.9

HM-266	*Juncus effusus* L.	1		0.23	3	0.0007	9.85714E-06	24043478.26	3.79121E-07	625130434.8
HM-267	*Melia toosendan* Sieb. *et* Zucc.	2		0.1	6	0.0006	8.57143E-06	27650000	3.2967E-07	718900000
HM-268	*Nelumbo nucifera* Gaertn.	1		0.12	5	0.0006	8.57143E-06	27650000	3.2967E-07	718900000
HM-269	*Aconitum kusnezoffii* Reichb.	3		0.39	1.2	0.0005	6.68571E-06	35448717.95	2.57143E-07	921666666.7
HM-270	*Oroxylum indicum* (L.) Vent.	1		0.1	3	0.0003	4.28571E-06	55300000	1.64835E-07	1437800000
HM-271	*Silybum mariamum* (L.) Gaertrn.	1, 3		5.5	For pharmaceutical preparation	For pharmaceutical preparation				
HM-272	*Chrysanthemum morifolium* Ramat.	1, 3		——	6	——				
HM-273	*Litsea cubeba* (Lour.) Pers.	2		——	30	——				
HM-274	*Sedum sarmentosum* Bunge	1		——	10	——				

HM-275	*Acanthopanax senticosus* (Rupr. et Maxim.) Harms	1	——	15	——
HM-276	*Fritillaria cirrhosa* D. Don	1	——	30	——
HM-277	*Areca catechu* L.	1	——	10	——
HM-278	*Rheum palmatum* L.	1	——	10	——
HM-279	*Paridis Rhizoma*	1	——	10	——
HM-280	*Angelica sinensis* (Oliv.) Diels	1	——	10	——
HM-281	*Kochia scoparia* (L.) Schrad.	1	——	10	——
HM-282	*Lycium chinense* Mill.	1	——	10	——
HM-283	*Eugenia caryophyllata* Thunb.	1	——	10	——

HM-284	*Angelica pubescens* Maxim. f. biserrata Shan *et* Yuan	1	——	10	——
HM-285	*Saposhnikovia divaricata* (Turcz.) Schischk.	1	——	10	——
HM-286	*Ligusticum sinense* Oliv.	1	——		——
HM-287	*Rhodiola crenulata* (Hook. f. et Thoms.) H. Ohba	1	——		——
HM-288	*Magnolia officinalis* Rehd. et Wils.	1	——		——
HM-289	*Polygonum cuspidatum* Sieb. et Zucc.	1	——		——
HM-290	*Zanthoxylum schinifolium* Sieb. *et* Zucc.	1	——		——

HM-291	*Coptis chinensis* Franch.	1	——	——
HM-292	*Citrus aurantium* L.	1	——	——
HM-293	*Allium tuberosu* Rottl. *ex* Spreng.	1	——	——
HM-294	*Prunus armeniaca* L. *var*. ansu Maxim.	1	——	——
HM-295	*Portulaca oleracea* L.	1	——	——
HM-296	*Dryopteris crassirhizoma* Nakai	1	——	——
HM-297	*Chaenomeles speciosa* (Sweet) Nakai	1	——	——
HM-298	*Sterculia lychnophora* Hance	1	——	——
HM-299	*Curcuma wenyujin* Y. H. Chen *et* C. Ling	1	——	——

HM-300	*Citrus reticulata* Blanco	1	——	——
HM-301	*Cistanche deserticoLa* Y. C. Ma	1	——	——
HM-302	*Amomum villosum* Lour.	1	——	——
HM-303	*Kaempferia galanga* L.	1	——	——
HM-304	*Dioscotea opposita* Thunb.	1	——	——
HM-305	*Polygonum orientale* L.	1	——	——
HM-306	*Pinus massoniana* Lamb.	1	——	——
HM-307	*Pseudostellaria heterophylla* (Miq.) Pax *ex* Pax *et* Hoffm.	1	——	——
HM-308	*Prunus persica* (L.) Batsch	1	——	——

HM-309	*Bolbostemma paniculatum* (Maxim.) Franque*t*	1	——	——
HM-310	*Terminalia chebula* Retz.	1	——	——
HM-311	*Prunella vulgaris* L.	1	——	——
HM-312	*Agrimonia pilosa* Ledeb.	1	——	——
HM-313	*Prunus humilis* Bge.	1	——	——
HM-314	*Rosa chinensis* Jacq.	1	——	——
HM-315	*Lycopus lucidus* Turcz. *var*. hirtus Regel	1	——	——
HM-316	*Anemarrhena asphodeloides* Bge.	1	——	——
HM-317	*Perilla frutescens* (L.) Britt.	1	——	——

HM-318	*Perilla frutescens* (L.) Britt.	1	——	——
HM-319	*Fritillaria thunbergii* Miq.	1	——	——
HM-320	*Hedysarum polybotrys* Hand. - Mazz.	3	——	——
HM-321	*Anemcme raddeana* Regel	1, 3	——	——
HM-322	*Trichosanthes kirilowii* Maxim.	1	——	——
HM-323	*Nelumbo nucifera* Gaertn.	1	——	——
HM-324	*Semiaquilegia adoxoides* (DC.) Makino	1	——	——
HM-325	*Arisaema erubescens* (Wall.) Schott	1	——	——
HM-326	*Acorus tatarinowii* Schott	1	——	——

HM-327	*Brucea javanica* (L.) Merr.	2	——	——
HM-328	*Aralia chinensis* L.	1	——	——
HM-329	*Piper longum* L.	1	——	——
HM-330	*Corydalis yanhusuo* W. T. Wang	1	——	——
HM-331	*Menispermum dauricum* DC.	1	——	——
HM-332	*Polygala tenuifolia* Willd.	1	——	——
HM-333	*Sinomenium acutum* (Thunb.) Rehd. *et* Wils.	1	——	——
HM-334	*Sargassum pallidum* (Turn.) C. Ag.	1	——	——
HM-335	*Platycladus orientalis* (L.) Franco	1	——	——

HM-336	*Nelumbo nucifera* Gaertn.	1	——	——
HM-337	*Benincasa hispida* (Thunb.) Cogn.	1	——	——
HM-338	*Campsis grandiflora* (Thunb.) K. Schum.	1	——	——
HM-339	*Ginkgo biloba* L.	1	——	——
HM-340	*Pinellia ternata* (Thunb.) Breit.	1	——	——
HM-341	*Rabdosia rubescens* (Hemsl.) Hara	1	——	——
HM-342	*Belamcanda chinensis* (L.) DC.	1	——	——
HM-343	*Dictamnus dasycarpus* Turcz.	1	——	——
HM-344	*Stellaria dichotoma* L. *var.* lanceolata Bge.	1	——	——

HM-345	*Glehnia littoralis* Fr. Schmidt *ex* Miq.	1	——	——
HM-346	*Quisqualis indica* L.	1	——	——
HM-347	*Ligusticum chuanxiong* Hort.	1	——	——
HM-348	*Descurainia sophia* (L.) Webb. *ex* Prantl.	1	——	——
HM-349	*Diospyros kaki* Thunb.	1	——	——
HM-350	*Punica granatum* L.	1	——	——
HM-351	*Ziziphus jujuba* Mill. *var*. spinosa (Bunge) Huex H. F. Chou	1	——	——
HM-352	*Euryale ferox* Salisb.	1	——	——
HM-353	*Polygonum bistorta* L.	1	——	——

HM-354	*Lindera aggregata* (Sims) Kos-term.	1	——	——
HM-355	*Citrus reticulata* Blanco	1	——	——
HM-356	*Polygonum multiflorum* Thunb.	1	——	——
HM-357	*Nelumbo nucifera* Gaertn.	1	——	——
HM-358	*Phellodendron chinense* Schneid.	1	——	——
HM-359	*Cetera cristoto* L.	1	——	——
HM-360	*Areca catechu* L.	1	——	——
HM-361	*Ligustrum lucidum* Ait.	1	——	——
HM-362	*Platycladus orientalis* (L.) Franco	1	——	——
HM-363	*Litchi chinensis* Sonn.	1	——	——

HM-364	*Ailanthus altissima* (Mill.) Swingle	1	——	——
HM-365	*Morus alba* L.	1	——	——
HM-366	*Ginkgo biloba* L.	1	——	——
HM-367	*Lonicera japoraca* Thunb.	1	——	——
HM-368	*Juglans regia* L.	1	——	——
HM-369	*Isatis indigotica* Fort.	1	——	——
HM-370	*Typha angustifolia* L.	1	——	——
HM-371	*Stemona sessilifolia* (Miq.) Miq.	1	——	——
HM-372	*Melia toosendan* Sieb. *et* Zucc.	1	——	——
HM-373	*Euodia rutaecarpa* (Juss.) Benth.	1	——	——

HM-374	*Spirodela polyrrhiza* (L.) Schleid.	1	——	——
HM-375	*Drynaria fortunei* (Kunze) J. Sm.	1	——	——
HM-376	*Solanum lyratum* Thunb.	1	——	——
HM-377	*Thalictrum foliolosum* DC.	1	——	——
HM-378	*Aralia chinensis* L.	1	——	——
HM-379	*Herba Artemisiae* Anomalae	2	——	——
HM-380	*Trollius chinensis* Bunge	2	——	——
HM-381	*Semen Zanthoxyli*	1	——	——
HM-382	*Hydnocarpus anthelmintica* Pierre	1	——	——
HM-383	*Solanum nigrum* L.	1	——	——
HM-384	*Nelumbo nucifera* Gaertn.	1	——	——

HM-385	*Gardenia jasminoides* Ellis	1	——	——
HM-386	*Alisma orientale* (Sam.) Juzep.	1	——	——

***(1---from Beijing Tongrentang Pharmacy store, China; 2---from Hebei Baicao Kangshen Pharmaceutical Co., Ltd, China; 3---from Anhui Bozhou Medicinal Materials Market, China)**
